# Evolution of Fish *Let-7* MicroRNAs and Their Expression Correlated to Growth Development in Blunt Snout Bream

**DOI:** 10.3390/ijms18030646

**Published:** 2017-03-16

**Authors:** Bo-Wen Zhao, Lai-Fang Zhou, Yu-Long Liu, Shi-Ming Wan, Ze-Xia Gao

**Affiliations:** 1 College of Fisheries, Key Lab of Agricultural Animal Genetics, Breeding and Reproduction of Ministry of Education/Key Lab of Freshwater Animal Breeding, Ministry of Agriculture, Huazhong Agricultural University, Wuhan 430070, China; zhaobowen@webmail.hzau.edu.cn (B.-W.Z.); 15171617087@163.com (L.-F.Z.); wansm0517@gmail.com (S.-M.W.); 2 Collaborative Innovation Center for Healthy Freshwater Aquaculture of Hubei Province, Hubei Provincial Engineering Laboratory for Pond Aquaculture, Wuhan 430070, China; 3 Department of Molecular and Cellular Biology, University of California Davis, Davis, CA 95616, USA; idrliu@ucdavis.edu

**Keywords:** fish, *Megalobrama amblycephala*, *let-7* miRNA, evolution, expression, growth development

## Abstract

The lethal-7 (*let-7*) miRNA, known as one of the first founding miRNAs, is present in multiple copies in a genome and has diverse functions in animals. In this study, comparative genomic analysis of *let-7* miRNAs members in fish species indicated that *let-7* miRNA is a sequence conserved family in fish, while different species have the variable gene copy numbers. Among the ten members including let-7a/b/c/d/e/f/g/h/i/j, the let-7a precursor sequence was more similar to ancestral sequences, whereas other *let-7* miRNA members were separate from the late differentiation of let-7a. The mostly predicted target genes of *let-7* miRNAs are involved in biological process, especially developmental process and growth through Gene Ontology (GO) enrichment analysis. In order to identify the possible different functions of these ten miRNAs in fish growth development, their expression levels were quantified in adult males and females of *Megalobrama amblycephala*, as well as in 3-, 6-, and 12-months-old individuals with relatively slow- and fast-growth rates. These ten miRNAs had similar tissue expression patterns between males and females, with higher expression levels in the brain and pituitary than that in other tissues (*p* < 0.05). Among these miRNAs, the relative expression level of *let-7a* was the highest among almost all the tested tissues, followed by *let-7b*, *let-7d* and *let-7c/e/f/g/h/i/j*. As to the groups with different growth rates, the expression levels of *let-7* miRNAs in pituitary and brain from the slow-growth group were always significantly higher than that in the fast-growth group (*p* < 0.05). These results suggest that *let-7* miRNA members could play an important role in the regulation of growth development in *M. amblycephala* through negatively regulating expression of their target genes.

## 1. Introduction

MicroRNAs (miRNAs) are a group of endogenous, short non-coding nucleotide sequences that play important roles in the post-transcriptional regulations of gene expression across organisms. Generally, mature miRNAs are approximately 18–25 nucleotide (nt) long while miRNA precursors (pre-miRNAs) are about 60–140 nt in length and with characteristic hairpin structures. As many studies have indicated that miRNAs have a complicated regulatory mechanism, including positive and negative correlations within and between miRNA’s upstream transcriptional factors or downstream targets. Important functions of miRNAs in gene regulation have been discovered in various physiological processes, including signal transduction, organ development [1], innate and adaptive immunity, and cell growth, differentiation, and apoptosis [2]. However, the rapid evolutionary dynamics in miRNAs, such as mutation, duplication, gene drift, and lineage-specific losses of paralogs, result in dozens of novel miRNAs emerging in the genomes of individual species of human, nematode, and fish [3]. Such novel miRNAs have identical or nearly identical mature sequences in contrast to the original sequence, which may acquire new targets and new functions, due to sequence variation of seed sequences at their 5’-ends [4,5,6]. Evidence suggests that the increasing number and diversification of miRNAs play an important role in biological evolution [7,8,9].

miRNAs are known for being deeply conservative in organic evolution; however, only a few miRNA families have been recently studied concerning their origin and evolution. Most studies have focused on the evolution of clusters or local duplication evolutionary patterns along with their host gene [6,10,11,12,13], since miRNAs are often co-transcribed with their host gene or evolve to form a cluster. According to miRBase, a cluster is defined as miRNAs with no more than 10 kb away from each other. The lethal-7 (*let-7*) miRNA family, known as one of the first two founding miRNAs, was discovered in *Caenorhabditis elegans* and played a crucial role in the heterochronic pathway that coordinates developmental timing [14,15]. The *let-7* miRNA family has been subsequently predicted and identified in a wide range of animal species, including vertebrates, ascidians, hemichordates, molluscs, annelids, and arthropods [16], with the sequence and function highly conserved from nematodes to primates [17]. As a critical regulator of gene expression, *let-7* family miRNAs are involved in multiple physiological processes, including temporal regulation [16,18], protein ubiquitylation [19], lens regeneration [20], signal regulation [21], cell proliferation and differentiation [2], sexual identity [22], and early development [23].

Fish are important in broad terms of ecology and food production [24]. With the development of high-throughput sequencing technologies in recent years, an increasing number of fish miRNAs have been reported by transcriptome analysis, such as 197 miRNAs from Japanese flounder (*Paralichthys olivaceus*) [25], 113 from common carp (*Cyprinus carpio*) [26], 282 from spot fork tail madtoms (*Ictalurus punctatus*) [27], and 764 in tilapia (*Oreochromis niloticus*) [28]. Among the identified mature miRNAs from these species, normally ten *let-7* miRNA members were found in each species, including *let-7a*, *let-7b*, *let-7c*, *let-7d*, *let-7e*, *let-7f*, *let-7g*, *let-7h*, *let-7i*, and *let-7j*. *Let-7* miRNAs have been reported to have diverse functions in fish, such as muscle formation [29], reproduction [30], and metamorphosis [31]; however, few in-depth studies have been carried out to investigate how *let-7* miRNAs have evolved in different fish species, as well as the relationship and the possible different roles among the ten *let-7* miRNAs members. Only one previous study reported the phylogenetic evolution of *let-7* miRNAs using data from four fish species and detected the expression levels of 10 *let-7* miRNAs during metamorphosis in *P. olivaceus* [31].

In this study, in order to investigate the evolutionarily conserved role of *let-7* miRNAs in fish, we performed comparative genomics, including multiple sequence alignment and an analysis of synteny in the orthology relationship to study the evolution of *let-7* miRNAs in different fish species. Through using one important commercial endemic fish species in Chinese freshwater polyculture system [32], blunt snout bream (*Megalobrama amblycephala*), we tried to elucidate the possible function of *let-7* miRNAs (*let-7a/b/c/d/e/f/g/h/i/j*) during different growth developmental stages in *M. amblycephala* individuals with relatively fast and slow growth rates through expression analysis. The evolution and expression analysis in this study will contribute to a better understanding of *let-7* miRNAs functions and evolution history, as well as their roles in regulating fish biological processes.

## 2. Results

### 2.1. Conserved Characteristics of Let-7 miRNAs in Fish

To characterize the evolution of the *let-7* miRNAs and analyze the relationship of their homologous, we collected all the let-7 precursor sequences of reported fish species from the miRBase. A total of 111 let-7 sequences were identified, with 23 single copies (Table S1). For the *I. punctatus*, 22 let-7 precursor sequences were found, including the specific copy of let-7a-7, let-7i-2, and let-7j-2, which were not found in other fish species.

**Figure 1:**
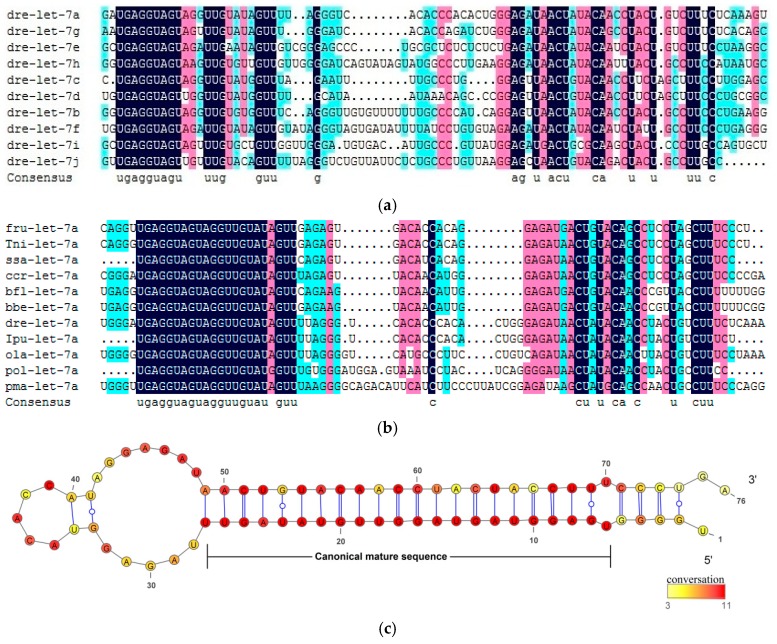
Sequence conservation in *let-7* miRNAs. (**a**) Multiple sequence alignments of let-7 paralogs precursor sequences (5′–3′); (**b**) Multiple sequence alignments of let-7a orthologs precursor sequences (5′–3′); and (**c**) Consensus secondary structure of the let-7a precursor in fish, the position of canonical mature sequence produced have indicated the hairpin structure in let-7a precursors. Different colors represent the sequence conservation at single base for (**a**,**b**).

The comprehensive analysis of *let-7* miRNAs homology relationship was conducted based on multiple sequence comparison assessment of synteny for both paralogs and orthologs. The sequence conservation of *let-7* miRNAs showed in multiple alignments of paralogs and orthologs precursor sequences (Figure 1). The results indicated that the 5′ arms of the *let-7* miRNAs precursor were highly conserved across paralogs and orthologs in fishes, while the 3′ arms had more nucleotide variation. However, two special cases were found in *I. punctatus* and *Branchiostoma floridae*, where conserved sequences of ipu-let-7j-2 and bfl-let-7b were located in the 3′ arm (Figure S1). It is worth noting that the bfl-let-7b conserved sequence was the reverse complementary sequence of *let-7b* sequence of other fish species, while its 5′ arm mature sequence had some nucleotides changes.

The highly conserved pattern among precursor sequences of *let-7* miRNAs suggests that the dominant mature miRNA was produced from the 5′ arm of let-7 precursors. It is found that the 5′ arm of let-7 orthologs was more conservative compared with paralogs, which means *let-7* miRNAs had experienced frequent duplication and nucleotide substitution events in its evolutionary history. According to the results of multiple sequence alignments against the paralogs and orthologs precursor sequences, the seed sequence of the 5′ arm was the same for all *let-7* miRNAs, while 3′ arm showed nucleotide diversity. Moreover, mature sequences of fish *let-7* miRNAs members were completely conservative in TGAGGTAGT and TTG and GTT (Figure S2).

### 2.2. The Evolution of Let-7 miRNAs in Fish

**Figure 2:**
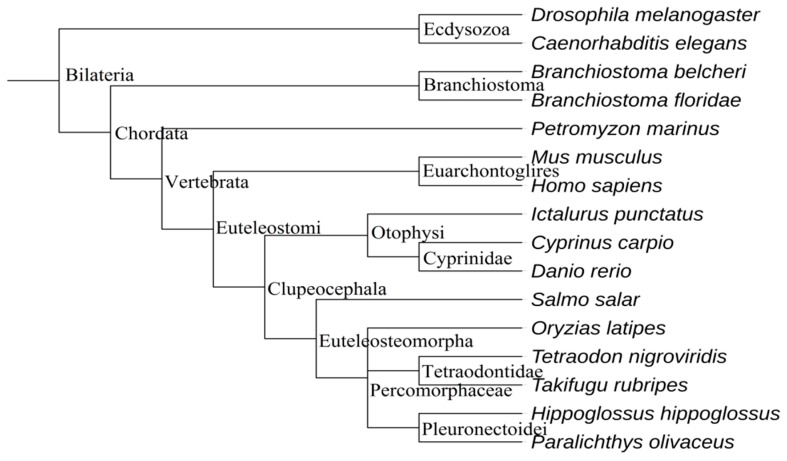
The genetic relationship for all the species of which *let-7* miRNAs sequences were collected in the present study.

All the sequences of *let-7* miRNAs members distributed in fish species, as well as five out-group species including *Homo sapiens*, *Mus musculus*, *Petromyzon marinus*, *Drosophila melanogaster*, and *Caenorhabditis elegans*, and two species considered to be the ancestors of fish (*Branchiostoma floridae* and *B. belcheri*), were collected in this study. The genetic relationship of these species was constructed as illustrated in Figure 2. Fish let-7 mature sequences always correspond to more precursor sequences. For example, *I. punctatus let-7a* has seven precursor sequences while human has just three.

**Figure 3:**
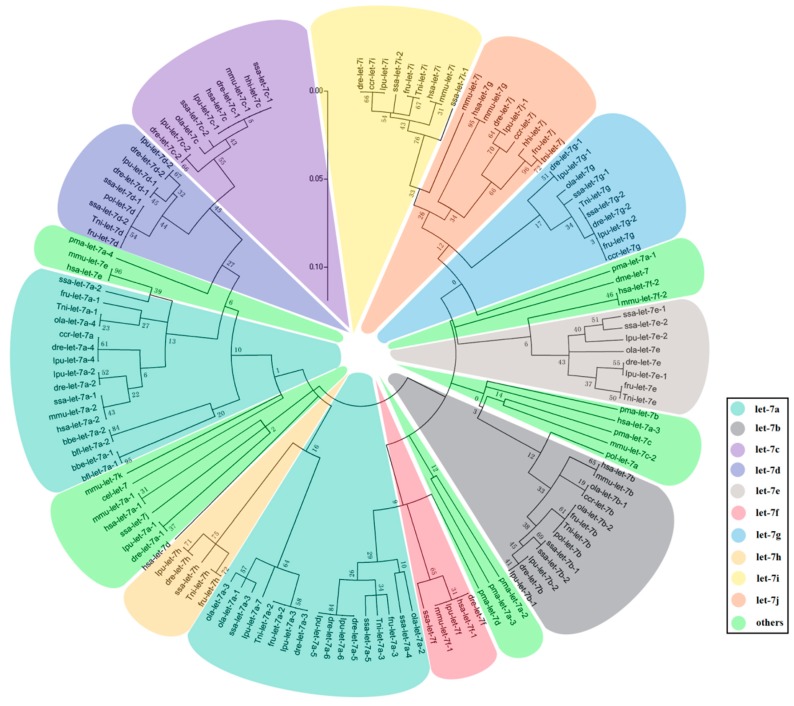
The phylogenic tree of *let-7* miRNAs in 16 species including nine fish species (*Danio rerio*-dre, *Oryzias latipes*-ola, *Fugu rubripes*-fru, *Tetraodon nigroviridis*-tni, *Ictalurus punctatus*-ipu, *Cyprinus carpio*-cca, *Paralichthys olivaceus*-pol, *Hippoglossus hippoglossus*-hhi, and *Salmo salar*-ssa), five out-group species (*Homo sapiens*-has, *Mus musculus*-mmu, *Petromyzon marinus*-pma, *Drosophila melanogaster*-dme, and *Caenorhabditis elegans*-cel) as well as two species considered to be the ancestors of fish (*Branchiostoma floridae*-bfl and *B. belcheri*-bbe).

To analyze the evolutionary mechanism of *let-7* miRNAs in fish, we constructed ML (maximum-likelihood) phylogenetic tree against all the fish let-7 precursor sequences, except ipu-let-7j-2. We considered that arm switching had been happened in precursor sequences of ipu-let-7j-2, which can be regarded as an accident event in let-7 evolution history. According to the result of the phylogenetic trees (Figure 3), the ancestral sequences of *let-7* miRNAs might have a mutation in two directions. One clade includes *let-7c*, *let-7d*, *let-7h*, and part of *let-7a* precursor sequences and the other is a big cluster including *let-7a*, *let-7b*, *let-7e*, *let-7f*, *let-7g*, *let-7i*, and *let-7j*. In all *let-7* miRNAs, *let-7c* and *let-7i* had the furthest genetic relationship with others in the two clades. *Let-7c* clustered with *let-7d*, *let-7a* and *let-7h* successively, and *let-7i* was closed to *let-7j*, *let-7g*, *let-7e*, *let-7b*, *let-7f*, and *let-7a* in tandem. It is noteworthy that each fish *let-7* miRNAs (except *let-7* miRNAs of *P. marinus*) as well as *B. floridae* and *B. belcheri let-7* miRNAs, are clustered in the same small branch.

Generally speaking, the miRNA cluster is divided into two main categories: either with another miRNA, or just clustered with its own homolog. Most of the miRNA clusters only exist in one form, whereas the form of let-7 clusters is more complicated. We have found two major clusters in fish: one includes two *let-7* miRNA in different combinations and the other is a form of mir-100/let-7a/mir-125 cluster (Table 1). Through the comparative analysis of *let-7* miRNA clusters in all fish species, we found that all the teleost *let-7* miRNAs have a mir-100/let-7a/mir-125 cluster, whereas mir-100 genes exist in the form of pma-mir-100-c/pma-mir-100-a/pma-mir-125 clusters in *P. marinus*. In *B. floridae*, there is only one special bfl-mir-100/bfl-let-7a-1/bfl-let-7b/bfl-mir-125a/bfl-mir-125b/bfl-let-7a-2 cluster in the genome.

### 2.3. Gene Ontology Enrichment Analysis

**Figure 4:**
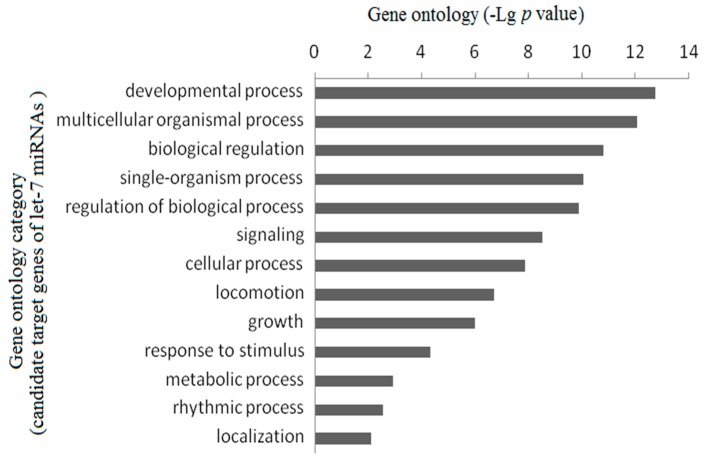
Gene ontology analysis of putative target genes of *let-7* miRNAs.

To organize the putative target genes of *let-7* miRNAs in hierarchical categories and elucidate the key biological process and molecular functions of *let-7* miRNAs may play in, we collected a total of 2378 candidate target genes by using Targetscan. The gene function enrichment evaluation showed that these genes were related to insulin-like growth factor binding, Wnt-activated receptor activity, Wnt-protein binding, insulin receptor substrate binding, growth factor activity and steroid hormone receptor activity, which play important role in growth development. Analysis of Gene Ontology biological process (GO-BP) terms indicates that most of the putative target genes are involved in developmental process, multicellular organismal process, biological regulation, single-organism process, and so on (Figure 4). This result suggests that *let-7* miRNAs may play a pivotal role in individual growth and development.

### 2.4. Expression Analysis of Let-7 miRNAs in Blunt Snout Bream

We have shown that mature let-7 sequences are highly conserved while its seed sequences are the same and likely share the same targeting properties across species. So, do fish *let-7* miRNAs also have the same function during growth development? We address this question by comparing the let-7 a/b/c/d/e/f/g/h/i/j in males and females of 12-month-old (immature) and 24-month-old (mature) *M. amblycephala*, as well as *let-7 a/b/c/d/e* miRNAs expression level in 3-, 6-, and 12-month-old fish with fast-growth and slow-growth groups. According to the small RNA transcriptome data, *M. amblycephala let-7* miRNAs have completely consistent mature sequences with zebrafish (Figure S3) and the *M. amblycephala let-7* miRNAs just have one or two base difference between each other. To distinguish the *let-7* miRNAs members better, we chose the TaqMan qRT-PCR assay to evaluate the expression of *let-7* miRNAs in *M. amblycephala*. Due to its remarkable functions in high specificity and sensitivity, highly homologous sequences in the same miRNA family can be distinguished accurately, even if there is only a one base difference, which makes this a suitable method for the study of the *let-7* miRNAs.

**Figure 5:**
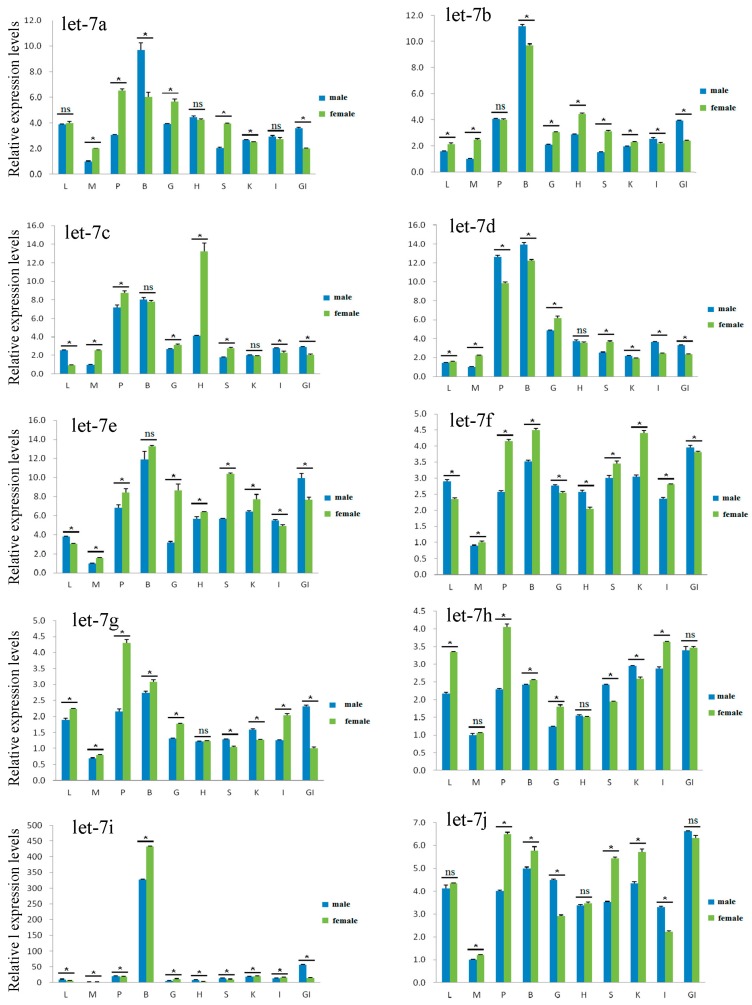
*Let-7* miRNAs expression in ten tissues of 24-month-old *M. amblycephala*. L, liver; M, muscle; P, pituitary; B, brain; G, gonad; H, heart; S, spleen; K, kidney; I, intestines; GI, gill. Statistical significance between females and males is indicated as follows: * *p* < 0.05; ns, not significant.

The results for *let-7 a/b/c/d/e/f/g/h/i/j* miRNAs expression in ten tissues of mature males and females *M. amblycephala* at 24 months old showed similar expression levels between males and females as to the same tissue in the most tested tissues (Figure 5); however, *let-7c* in heart and *let-7i* in gill had a more than three-fold gap between males and females (*p* < 0.05). The *let-7* miRNAs expression between male and female individuals followed the same expression pattern in different tissues, with the expression level of *let-7* miRNAs in brain and pituitary being significantly higher compared with that in other tissues (*p* < 0.05). The relative expression level of *let-7a* showed the highest value in almost all the tested tissues among the ten miRNAs, followed by *let-7b*, *let-7d*, and *let-7c/e/f/g/h/i/j*.

**Figure 6:**
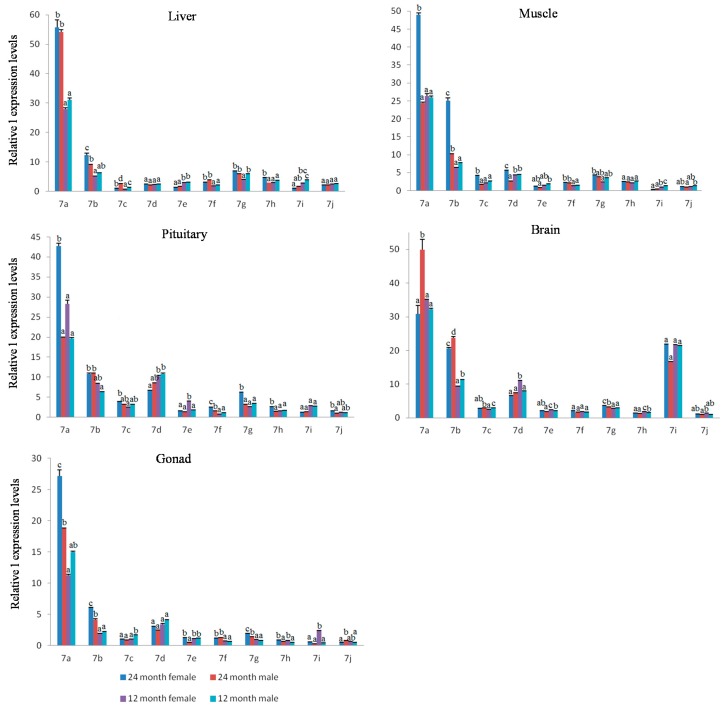
The relative expression levels of *let-7* miRNAs (*let-7a/b/c/d/e/f/g/h/i/j*) in liver, muscle, pituitary, brain and gonad tissues at 12- and 24-months-old *M. amblycephala* (females and males). Values with the same letter mean no significant difference (*p* > 0.05) for each miRNA in females and males at 12- and 24-months-old *M. amblycephala*.

In immature *M. amblycephala* at 12 months old, expression levels were analyzed in five tissues of male and female individuals (Figure S4). The results showed that *let-7* miRNAs expression patterns in tissues of immature males and females were almost the same as that in sexually mature individuals, with ten *let-7* miRNA members having the highest expression in brain and pituitary. The results for 12-month-old and 24-month-old individuals showed that *let-7c/d/e/g/h/i/j* had a similar expression levels in the tissues of *M. amblycephala* from these two stages (Figure 6), whereas *let-7a/b/f* had a higher expression level in 24-month-old males and females of *M. amblycephala* (*p* < 0.05).

**Figure 7:**
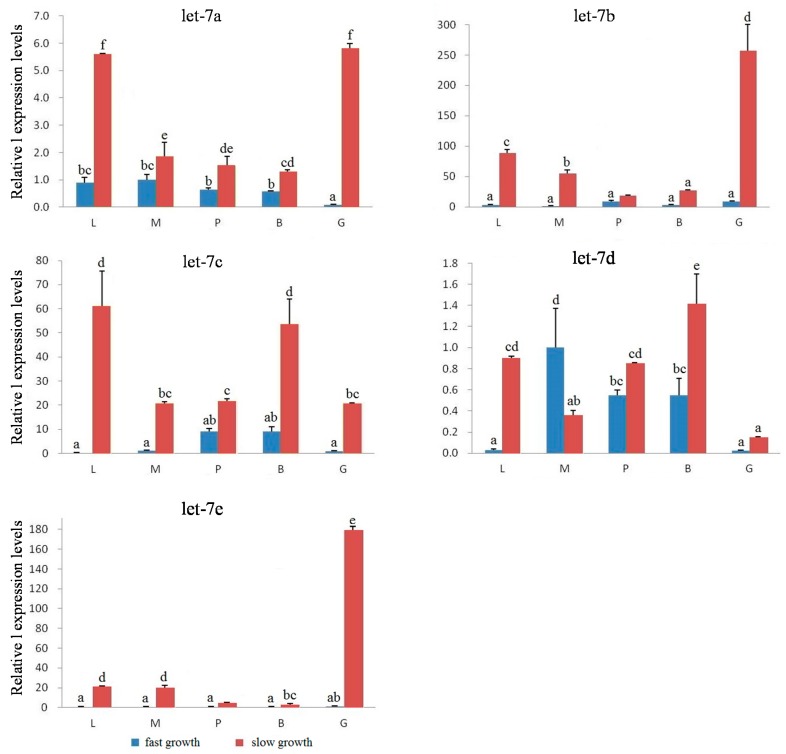
The relative expression levels of *let-7a/b/c/d/e* miRNAs in slow- and fast-growth groups of *M. amblycephala* at 3-month-old stage. L, liver; M, muscle; P, pituitary; B, brain; G, gonad. Values with the same letter mean no significant difference (*p* > 0.05).

According to the expression results of 12- and 24-month-old individuals, we selected five miRNAs including let-7 a/b/c/d/e, which showed significant differences or high expression levels compared with other *let-7* miRNAs, to further explore the possible regulation functions of the key *let-7* miRNA for *M. amblycephala* growth development. In 3-month-old *M. amblycephala*, *let-7 a/b/c/d/e* expressions were tested in the fast- and slow-growth groups. The results found that all five *let-7* miRNAs showed significant differences between fast- and slow-growth individuals as to the same tissue (*p* < 0.05), with significantly higher expression in the slow-growth group (*p* < 0.05; Figure 7), except *let-7d* in muscle. The expression differences of five *let-7* miRNAs were particularly significant in liver, muscle and gonad tissues, with differences up to dozens or even hundreds of times between fast- and slow-growth groups. Among these five *let-7* miRNAs, *let-7a* and *let-7b* had relatively higher expression levels than the other three miRNAs (Figure S5).

The *let-7* miRNAs quantitative analysis results of 6-month-old *M. amblycephala* showed that *let-7* miRNAs had similar expression patterns in tested tissues between fast- and slow-growth groups, with relatively higher expression in brain and pituitary (*p* > 0.05; Figure S6). The *let-7* miRNAs expression at 12-month-old individuals from fast- and slow-growth groups showed that the expression in the fast-growth group was slightly higher than that in the slow-growth group in liver, muscle and gonad, but not in the pituitary and brain (Figure S7).

**Figure 8:**
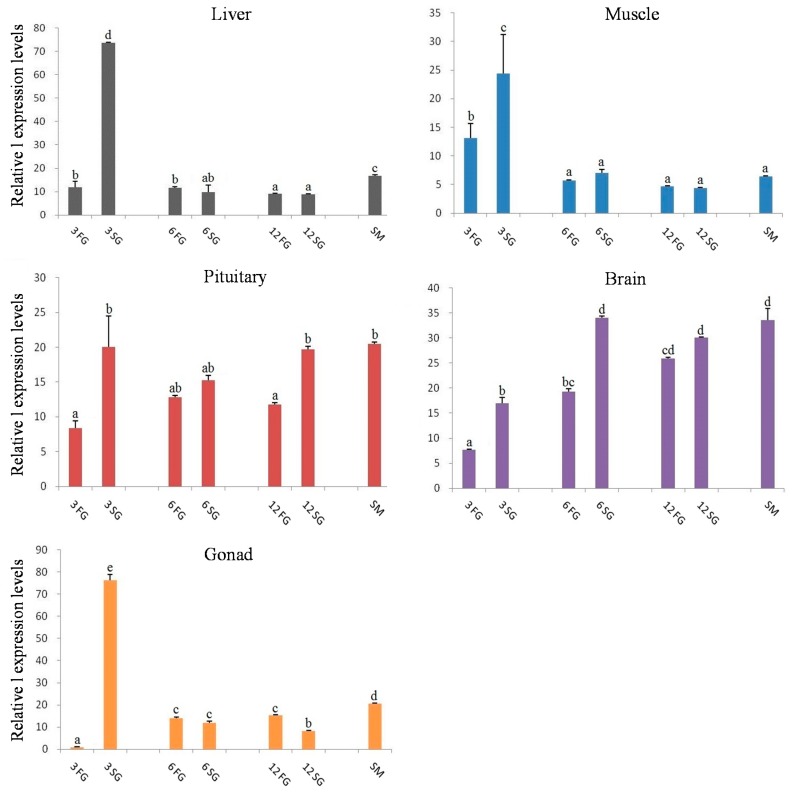
The relative expression levels of *let-7a* in different tissues from different growth stages of *M. amblycephala*. 3FG, fast-growth individuals at 3 months old; 3SG, slow-growth individuals at 3 months old; 6FG, fast-growth individuals at 6 months old; 6SG, slow-growth individuals at 6 months old; 12FG, fast-growth individuals at 12 months old; 12SG, slow-growth individuals at 12 months old; SM, mature individuals. Values with the same letter mean no significant difference (*p* > 0.05).

Relative expression of *let-7* miRNAs in 3-, 6-, and 12-month-old *M. amblycephala* were compared in the same tissue (Figure 8). The results showed that in liver, muscle, pituitary, brain, and gonad, *let-7* miRNAs had a significantly higher expression level in the slow-growth group compared with the fast-growth group at 3 months, but not in other growth stages between slow-growth and fast-growth groups. However, as to pituitary and brain tissues, the expression levels of *let-7* miRNAs in the slow-growth group were significantly higher than that in the fast-growth group for all the three growth stages, with *let-7a/b/d* having relatively higher expression than that of *let-7c* and *let-7e* (Figure S8). Moreover, it was also found that in most cases, the *let-7* expression in sexually mature *M. amblycephala* was slightly higher than either fast-growth or slow-growth individuals at 3-, 6-, and 12-month-old *M. amblycephala* in most tissues.

## 3. Discussion

The evolutionary process of miRNA families always occurs along with locus mutation, which can lead to a series of influence, such as gene structure and function change. As the biggest miRNA family, *let-7* miRNAs have experienced frequent genome duplication, losses, rearrangements and transpositions in their evolution history [7,33,34,35]. The formation of multiple *let-7* miRNA members is mainly due to miR gene duplicates usually reserved after genome duplication events, which may cause an increasing number of homologs in the end [8].

Independent tandem duplication events can result in increasing numbers of gene families; however, duplication of the entire genome is the major driver to promote genetic evolution and phenotypic complexity. In this study, it was found that fish let-7 family members have more copy numbers compared with tetrapods, and this phenomenon is in accordance with a previous study that found ray-finned fish gene families always have two copies, whereas tetrapods just have one [36,37]. In fact, some phylogenomic studies have found that the entire genomes of most vertebrates have experienced two rounds (2R) of duplication in their evolution, but the stem lineage of ray-finned (actinopterygian) fishes has been subject to an extra fish-specific genome duplication (FSGD) event in approximately 350 Ma [38,39,40]. Therefore, it was considered as the reason for genomic complexity and biological diversity of teleost. Interestingly, in other research, *let-7* miRNA was found to play a special role in their primary transcript process and they can enhance primary transcript processing by binding at the 3′-end [41]. This unique biological process may result in frequent replication and extra copies, which also could be deemed as an important reason for the formation of complex *let-7* miRNA members in fish.

The result of multiple alignments has shown that the mature sequence of *let-7* miRNA precursor sequences was found in the 5′ instead of 3′ arm in most previous studies [12]; however, it can be a normal phenomenon considering the complementary pairing relations between the 5′ and 3′ arm sequences in the hairpin structure, and the mutation is random. In the study of mir-155 evolution, the 5′ arm was also highly conserved compared with the 3’ arm sequences [42]. In this study, ipu-let-7j-2 and bfl-let-7b were identified as special cases in the evolution of *let-7* miRNA, from which we speculate that ipu-let-7j-2 had experienced arm switching in precursor sequences since the mature sequence appeared in the 3′ instead of 5′ arm. However, bfl-let-7b mature sequence is the complementary sequence of let-7b in other species, which means mutations to the 5′ arm of bfl-let-7b have occurred in evolution. The existence of bfl-let-7b also confirmed the randomness of the conservative area in the let-7 gene 5′ or 3′ arms. Even though let-7 gene has experienced frequent duplication events and nucleotide changes in its long evolution history, the seed sequences of all let-7 family members was completely conserved in fish. Considering that the miRNAs functions in gene regulation is through binding the seed sequence and target genes 3′ non-coding regions, we infer that let-7 family members may have the same or similar functions in fish. Let-7 family has been confirmed as a temporal regulation gene in animal development; therefore, it is possible that let-7 members play different roles at different stages of fish development.

According to the ML phylogenetic tree we constructed, *let-7a* is one of two branches separated from an ancestral *let-7* gene sequence, which means *let-7a* was more similar to the ancestor’s sequence. It was easy to find the process of *let-7a* duplication and differentiation throughout the whole let-7 family evolution history. In the paralogs, *let-7i*, *let-7j*, *let-7g*, *let-7e*, *let-7f*, and *let-7b* were derived from earlier differentiation event, while the appearances of let-7d, let-7c, and let-7h were relatively late. Thus, the ancestral sequences of let-7a can be considered as the ancestral sequences of let-7 family. The close relationship of let-7 sequences from *B. floridae*, *B. belcheri* and fish also confirmed the genetic relationship of amphioxus and fish. However, it needs to note that pma-let-7 miRNAs are independent in evolution after *let-7a* differentiation, which showed that pma-let-7b, pma-let-7c and pma-let-7d occurred after differentiation of gnathostome and agnatha. As the tightly linked physical distance, cluster genes usually co-evolve and duplicate together, which enables the study their evolution by analyzing their relationships. We found no mir-100/let-7a/mir-125 cluster in *P. marinus* although it appeared in other fish, which suggests that the mir-100/let-7a/mir-125 cluster was also formed after the differentiation of agnatha and gnathostomata. Both phenomena indicated that *let-7* miRNA evolution may have been divided following the differentiation of agnatha and gnathostomata.

The *let-7* miRNAs had been reported to be widely expressed in various animal tissues. For example, in the domesticated silkmoth (*Bombyx mori*), strong signal in the head, moderate signals in the body wall, midgut, gonad, and malpighian tubule were found, whereas a weak positive signal was seen in the anterior and posterior silk glands [43]; and in *Homo sapiens*, expression was seen in the brain, heart, kidneys, liver, lungs, trachea, bone marrow, colon, small intestine, spleen, stomach, and thymus [16]. As to fish species, Fu et al. [31] found that *let-7* miRNAs were widely expressed in adult tissues and highly expressed in the brain, heart and stomach in *P. olivaceus*. The results from the present study also showed that *let-7a/b/c/d/e/f/g/h/i/j* miRNAs were widely expressed in all the tested tissues in adult *M. amblycephala* (24 months old). The *let-7a/b/d* had relatively higher expression than that of other *let-7* miRNAs, and all *let-7* miRNAs had relatively higher expression in brain and pituitary tissues. Moreover, the expression levels of *let-7c/d/e/g/h/i/j* at 12 and 24 monthss had similar expression patterns in the tested five tissues, whereas *let-7a/b/f* had a higher expression level in 24-month-old females and males than in 12-month-old *M. amblycephala* (*p* < 0.05). These findings indicate that *let-7* miRNAs could play diverse roles in a variety of metabolic processes; thus, higher expression levels of *let-7* miRNAs might indicate that they play more important roles in regulating the development or metabolism of the related tissues at different development stages in fish species.

Since discovered as an essential developmental gene in *C. elegans*, *let-7* miRNAs have been confirmed to be involved in a broad variety of biological functions in many animals. Due to the importance of growth rate for commercial breeds, there is great interest in gaining a better understanding of the networks of expressed genes and the biological pathways controlling growth rate. Comparative analyses of expression profiles are useful in identifying the molecular differences between divergent muscle phenotypes. In mammals, *let-7b*, as a critical miRNA, has been demonstrated to be involved in the regulation of growth hormone receptor (*GHR*) and plays a critical role in regulating skeletal muscle growth via let-7b-mediated *GHR* expression in deletion-type dwarf chickens [44]. In pig, it has also been proved that *let-7a* and *let-7f* were involved in its skeletal muscle development [45]; however, no related studies have indicated that the expressions of *let-7* miRNAs were related to fish growth as well as which *let-7* miRNAs members would be more important. In the present study, the expression results of five *let-7* miRNAs from 3-months-old *M. amblycephala* showed significantly higher expression levels in the slow-growth group compared with the fast-growth group for all tested tissues. As to the 6- and 12-months-old individuals, the expression levels of *let-7* miRNAs in pituitary and brain from the slow-growth group were also significantly higher than that in the fast-growth group. Among these five miRNAs, *let-7a/b/d* had relatively higher expression levels than that of *let-7c* and *let-7e*. As miRNAs always negatively regulate gene expression at post-transcriptional level by complementary binding to the 3’-UTR of target messenger RNAs (mRNAs) and causing mRNA cleavage or translation blockage [46], it is suggested that all the upstream and downstream genes in related tissues as well as its receptor genes may be under the regulation of *let-7* miRNAs in *M. amblycephala*. These results suggest that *let-7* miRNA members could play an important role in the regulation of growth development in fish species, with their target genes mainly expressed in the pituitary and brain tissues. The GO analysis also indicated that the putative target genes of *let-7* miRNAs are involved in growth and development process. The functions of *let-7* miRNAs in other fish species needs be further studied.

## 4. Materials and Methods

### 4.1. Evolutionary Analysis of Let-7 miRNA

For the analysis of let-7 evolution, we collected all the let-7 information from fish, including all members of *let-7* miRNAs, annotations, corresponding species, precursor sequences and miRNA/miRNA* complexes from different species in the miRBase database release 21 (http://www.mirbase.org/index.shtml). All *let-7* miRNAs sequences were gathered from 16 species, including nine fish species (*Danio rerio*-dre, *Oryzias latipes*-ola, *Fugu rubripes*-fru, *Tetraodon nigroviridis*-tni, *Ictalurus punctatus*-ipu, *Cyprinus carpio*-cca, *Paralichthys olivaceus*-pol, *Hippoglossus hippoglossus*-hhi, and *Salmo salar*-ssa), five out-group species (*Homo sapiens*-has, *Mus musculus*-mmu, *Petromyzon marinus*-pma, *Drosophila melanogaster*-dme, and *Caenorhabditis elegans*-cel), as well as two species considered to be the ancestors of fish (*Branchiostoma floridae*-bfl and *B. belcheri*-bbe). All the sequences are attached in Table S1. The distribution and genetic relationship of these species were analyzed by the Taxonomy Tools of the NCBI (http://www. ncbi.nlm.nih.gov/taxonomy/) [42].

To analyze sequence conservation, we performed multiple sequence alignment of *let-7* miRNAs precursor sequences with Clustal X2.0 [47] and the alignment result was colored by DNAman6.0 [42]. The fasta alignments were performed using BioEdit according to the result of Stockholm alignment. We constructed consensus sequences by taking the most abundant base for each column, and consensus secondary structure was predicted by Mfold version2.3 (http://unafold.rna.albany.edu/?q=mfold/RNA-Folding-Form2.3) [48]. VARNA 3.93 was used to visualize the consensus miRNA structure [49]. Let-7 evolution analysis and the confirmation of ancestral sequence were performed with MEGA 5.0 by constructed phylogenetic trees, under the condition of 1000 bootstrap resampling using Maximum-Likehoood (ML) method [50].

### 4.2. Prediction and Gene Ontology(GO) Analysis of Let-7 miRNAs Target Genes

The canditade target genes of *let-7* miRNAs were predicted using the Targetscan method. Targetscan is one of most widely used algorithm in miRNA target gene prediction, and this algorithm put forward the concept of “seed area” [51]. DAVID 6.8 was used to perform GO analysis on putative target genes [52,53], focusing on the Gene Ontology biological process (GO-BP) terms and Gene Ontology molecular function (GO-MF) term of *let-7* miRNAs. Significane threshold *p* was set as 0.05, and all the *p*-value are Benjamin *p*-value from DAVID.

### 4.3. Expression Analysis of Let-7 miRNA

All experimental fish *M. amblycephala* were collected from the Tuanfeng Fish Breeding Base of College of Fisheries, Huazhong Agricultural University. All experimental procedures involving fish were approved by the institution’s animal care and use committee of the Huazhong Agricultural University. After two weeks of acclimation and feed, tissue samples were collected from healthy individuals at 24 months old (sexually mature) and 12 months old (sexually immature) *M. amblycephala* (female and male groups), as well as 3-month-old, 6-month-old, and 12-month-old *M. amblycephala* with fast-growth and slow-growth groups; each stage from each group included three individuals. The phenotypic data of these two groups are showed in Table S2. Before tissue collection, experimental fish were placed in 100 mg/L concentration of tricaine methanesulfonate (MS-222) water for anesthesia [32]. We collected 10 fresh tissues from 24-month-old *M. amblycephala* individuals, including liver (L), muscle (M), pituitary (P), brain (B), gonad (G), heart (H), spleen (S), kidney (K), intestines (I), and gill (GI). In 3-month-old, 6-month-old and 12-month-old *M. amblycephala*, five tissues (L, M, P, B, and G) were collected to detect gene expression. All the samples obtained were frozen in liquid nitrogen immediately and then transferred to −80 °C before total RNA extraction.

Total RNA of each sample were extracted using Trizol reagent (Invitrogen, Carlsbad, CA, USA) according to the manufacturer’s recommendations. The qualitative and quantitative evaluation of RNA was checked on the NanoDrop 2000 (Thermo Fisher Scientific, Waltham, MA, USA). Then, 10 ng of total RNA was reverse transcribed into complementary DNA (cDNA) with TaqMan^®^ MicroRNA Assays (Applied Biosystems, Waltham, MA, USA) and TaqMan^®^ MicroRNA Reverse Transcription Kit (Applied Biosystems). Each reaction contained 0.15 μL of 100 mM dNTP’s (with dNTP), 1 μL of MultiScribeTM Reverse Transcription 50 U/μL, 1.5 μL of 10× Reverse Transcription Buffer, 0.19 μL of RNase Inhibitor 20 U/L, 4.16 μL of Nuclease-free water, 5 μL of RNA template, and 3 μL of Reverse Transcription primer were added to a final volume of 15 μL. The mixed samples were incubated at the following conditions: 30 min at 16 °C, 30 min at 42 °C, 5 min at 85 °C, and 4 °C. Products from RT reactions were used for real-time PCR directly.

Real-time PCR was performed using TaqMan MicroRNA Assays and TaqMan Universal PCR Master Mix II, no AmpErase UNG on a QuantStudio™ 6 Flex real-time PCR System. For quantification, 1.33 μL of original PCR product was mixed with 1.0 μL TaqMan^®^ MicroRNA Assays (20×), 10 μL TaqMan Universal PCR Master Mix II and 7.67 μL nuclease-free water in each 20 μL volume reaction. Standard amplification conditions were as follow: an initial 10 min pre-incubation at 95 °C followed by 40 cycles of 95 °C for 15 s and 60 °C for 1 min. The miR-26, 18S rRNA and β-actin were all used as reference genes in quantification analysis of different tissues from sexually mature females and males. Then the relative stability measure (*M*) of the reference genes was calculated by GeNorm (https://genorm.cmgg.be/) [54] as described in our previous studies [55]. The value *M* represents an average pairwise variation of a reference gene with all other reference genes and a lower *M* value corresponds to the higher expression stability. According to this rule, miR-26 with the *M* value of 0.772, lower than 18S rRNA (0.914) and β-actin (0.787), was employed as the endogenous control gene for all the analysis samples [23].

### 4.4. Data Analysis

The gene expression changes determined in the quantitative real-time PCR experiment were quantified based on the comparative *C*t method (2^−ΔΔ*C*t^ formula). Statistical analysis of miRNA expression data was performed using the SPSS 22 software, and statistical significance was analyzed by single-factor analysis of variance and two-tailed Student’s *t*-test (confidence interval 95%). Statistical significance is indicated as follows: * *p* < 0.05; N.S., not significant.

## 5. Conclusions

This study showed that the let-7 family is a sequence and function conserved family in fish, which has a long evolutionary history with frequent genome duplication, losses, rearrangements, and transposition events. The evolution of the let-7 family is random and chaotic process; hence, different species have highly variable gene copy numbers. The *let-7a* precursor sequence was more similar to ancestral sequences, whereas other *let-7* miRNAs were separate from the late differentiation of *let-7a*. *Let-7* miRNAs have shown the stage- and tissue-dependent expression patterns in *M. amblycephala* growth development. Our results confirmed that *let-7* miRNAs had a similar expression pattern in *M. amblycephala* male and female individuals, and the expression results suggest that *let-7* miRNAs may play an important role in the regulation of growth development in fish species, with *let-7a* and *let-7b* being most important.

## Figures and Tables

**Table 1:** The typical let-7 clusters identified in this study.

*Danio rerio*	*Oryzias latipes*	*Fugu rubripes*	*Petromyzon marinus*	*Tetraodon nigroviridis*	*Branchiostoma floridae*
dre-let-7a-1/dre-let-7f	ola-let-7a-1/ola-let-7b-1				
dre-mir-100-1/dre-let-7a-2/dre-mir-125b-1		fru-mir-100/fru-let-7a-1/fru-mir-125b		tni-mir-100/tni-let-7a-1/tni-mir-125b	
dre-let-7a-3/dre-let-7b	ola-let-7a-3/ola-let-7b-2		pma-let-7a-3/pma-let-7d		
dre-mir-100-2/dre-let-7a-4/dre-mir-125b-2	ola-mir-100-2/ola-let-7a-4/ola-mir-125b-1				
dre-let-7e/dre-let-7a-5	ola-let-7e/ola-let-7a-2	fru-let-7e/fru-let-7a-3			
dre-mir-99-1/dre-let-7c-1/dre-mir-125c	ola-mir-99/ola-let-7c/ola-mir-125b-2				
dre-mir-99-2/dre-let-7c-2					
dre-let-7g-2/dre-let-7h		fru-let-7g/fru-let-7h		tni-let-7g/tni-let-7h	
			pma-mir-100c/pma-mir-100a/pma-mir-125		
					bfl-mir-100/bfl-let-7a-1/bfl-let-7b/bfl-mir-125a/bfl-mir-125b/bfl-let-7a-2

## Supplementary Materials

Supplementary materials can be found at www.mdpi.com/1422-0067/18/3/646/s1.

## References

[1] Kumar M.S., Erkeland S.J., Pester R.E., Chen C.Y., Ebert M.S., Sharp P.A., Jacks T. (2008). Suppression of non-small cell lung tumor development by the *let-7* microRNA family. Proc. Natl. Acad. Sci. USA.

[2] Ecsedi M., Grosshans H. (2013). LIN-41/TRIM71: Emancipation of a miRNA target. Genes Dev..

[3] De Wit E., Linsen S.E., Cuppen E., Berezikov E. (2009). Repertoire and evolution of miRNA genes in four divergent nematode species. Genome Res..

[4] Marco A., Hooks K., Griffiths-Jones S. (2012). Evolution and function of the extended miR-2 microRNA family. RNA Biol..

[5] Nachtigall P.G., Dias M.C., Pinhal D. (2014). Evolution and genomic organization of muscle microRNAs in fish genomes. BMC Evol. Biol..

[6] Wu S., Aksoy M., Shi J., Houbaviy H.B. (2014). Evolution of the miR-290-295/miR-371-373 cluster family seed repertoire. PLoS ONE.

[7] Hertel J., Lindemeyer M., Missal K., Fried C., Tanzer A., Flamm C., Hofacker I.L., Stadler P.F. (2006). Students of Bioinformatics Computer Labs 2004 and 2005. The expansion of the metazoan microRNA repertoire. BMC Genom..

[8] Heimberg A.M., Sempere L.F., Moy V.N., Donoghue P.C.J., Peterson K.J. (2008). MicroRNAs and the advent of vertebrate morphological complexity. Proc. Natl. Acad. Sci. USA.

[9] Wheeler B.M., Heimberg A.M., Moy V.N., Sperling E.A., Holstein T.W., Heber S., Peterson K.J. (2009). The deep evolution of metazoan microRNAs. Evol. Dev..

[10] Hertel J., Bartschat S., Wintsche A., Otto C., Stadler P.F., Students of the Bioinformatics Computer Lab (2012). Evolution of the *let-7* microRNA Family. RNA Biol..

[11] Bhuiyan S.S., Kinoshita S., Wongwarangkana C., Asaduzzaman M., Asakawa S., Watabe S. (2013). Evolution of the myosin heavy chain gene *MYH14* and its intronic microRNA miR-499: Muscle-specific miR-499 expression persists in the absence of the ancestral host gene. BMC Evol. Biol..

[12] Desvignes T., Contreras A., Postlethwait J.H. (2014). Evolution of the miR199-214 cluster and vertebrate skeletal development. RNA Biol..

[13] Quah S., Holland P.W. (2015). The Hox cluster microRNA miR-615: A case study of intronic microRNA evolution. EvoDevo.

[14] Lee R.C., Feinbaum R.L., Ambms V. (1993). The *C. elegans* heterochronic gene *lin-4* encodes small RNAs with antisense complementarity to *lin-14*. Cell.

[15] Reinhart B.J., Slack F.J., Basson M., Pasquinelli A.E., Bettinger J.C., Rougvie A.E., Horvitz H.R., Ruvkun G. (2000). The 21-nucleotide *let-7* RNA regulates developmental timing in *Caenorhabditis elegans*. Nature.

[16] Pasquinelli A.E., Reinhart B.J., Slack F., Martindale M.Q., Kuroda M.I., Maller B., Hayward D.C., Ball E.E., Degnan B., Müller P. (2000). Conservation of the sequence and temporal expression of *let-7* heterochronic regulatory RNA. Nature.

[17] Lin Y.C., Hsieh L.C., Kuo M.W., Yu J., Kuo H.H., Lo W.L., Lin R.J., Yu A.L., Li W.H. (2007). Human *TRIM71* and its nematode homologue are targets of *let-7* microRNA and its zebrafish orthologue is essential for development. Mol. Biol. Evol..

[18] Roush S.F., Slack F.J. (2009). Transcription of the *C. eleganslet-7* microRNA is temporally regulated by one of its targets, *hbl-1*. Dev. Biol..

[19] Rybak A., Fuchs H., Hadian K., Smirnova L., Wulczyn E.A., Michel G., Nitsch R., Krappmann D., Wulczyn F.G. (2009). The let-7 target gene mouse *lin-41* is a stem cell specific E3 ubiquitin ligase for the miRNA pathway protein Ago2. Nat. Cell Biol..

[20] Nakamura K., Maki N., Trinh A., Trask H.W., Gui J., Tomlinson C.R., Tsonis P.A. (2010). miRNAs in newt lens regeneration: Specific control of proliferation and evidence for miRNA networking. PLoS ONE.

[21] Zhao Y., Deng C., Wang J., Xiao J., Gatalica Z., Recker R.R., Xiao G.G. (2011). Let-7 family miRNAs regulate estrogen receptor α signaling in estrogen receptor positive breast cancer. Breast Cancer Res. Treat..

[22] Fagegaltier D., König A., Gordon A., Lai E.C., Gingeras T.R., Hannon G.J., Shcherbata H.R. (2014). A genome-wide survey of sexually dimorphic expression of *Drosophila* miRNAs identifies the steroid hormone-induced miRNA let-7 as a regulator of sexual identity. Genetics.

[23] Ouchi Y., Yamamoto J., Iwamoto T. (2014). The heterochronic genes *lin-28a* and *lin-28b* play an essential and evolutionarily conserved role in early zebrafish development. PLoS ONE.

[24] Bizuayehu T.T., Babiak I. (2014). MicroRNA in Teleost Fish. Genome Biol. Evol..

[25] Fu Y., Shi Z., Wu M., Zhang J., Jia L., Chen X. (2011). Identification and differential expression of microRNAs during metamorphosis of the Japanese flounder (*Paralichthys olivaceus*). PLoS ONE.

[26] Zhu Y.P., Xue W., Wang J.T., Wan Y.M., Wang S.L., Xu P., Zhang Y., Li J.T., Sun X.W. (2012). Identification of common carp (*Cyprinus carpio*) microRNAs and microRNA-related SNPs. BMC Genom..

[27] Xu Z., Chen J., Li X., Ge J., Pan J., Xu X. (2013). Identification and characterization of microRNAs in channel catfish (*Ictalurus punctatus*) by using Solexa sequencing technology. PLoS ONE.

[28] Xiao J., Zhong H., Zhou Y., Yu F., Gao Y., Luo Y., Tang Z., Guo Z., Guo E., Gan X. (2014). Identification and characterization of microRNAs in ovary and testis of Nile tilapia (*Oreochromis niloticus*) by using Solexa sequencing technology. PLoS ONE.

[29] Johnston I.A., Lee H.T., Macqueen D.J., Paranthaman K., Kawashima C., Anwar A., Kinghorn J.R., Dalmay T. (2009). Embryonic temperature affects muscle fibre recruitment in adult zebrafish: Genome-wide changes in gene and microRNA expression associated with the transition from hyperplastic to hypertrophic growth phenotypes. J. Exp. Biol..

[30] Bizuayehu T.T., Lanes C.F., Furmanek T., Karlsen B.O., Fernandes J.M., Johansen S.D., Babiak I. (2012). Differential expression patterns of conserved miRNAs and isomiRs during Atlantic halibut development. BMC Genom..

[31] Fu Y., Shi Z., Wang G., Zhang J., Li W., Jia L. (2013). Expression of *let-7* microRNAs that are involved in Japanese flounder (*Paralichthys olivaceus*) metamorphosis. Comp. Biochem. Physiol. B Biochem. Mol. Biol..

[32] Yi S., Gao Z.X., Zhao H., Zeng C., Luo W., Chen B., Wang W.M. (2013). Identification and characterization of microRNAs involved in growth of blunt snout bream (*Megalobrama amblycephala*) by Solexa sequencing. BMC Genom..

[33] Yuan Z., Sun X., Jiang D., Ding Y., Lu Z., Gong L., Liu H., Xie J. (2010). Origin and evolution of a placental-specific microRNA family in the human genome. BMC Evol. Biol..

[34] Nozawa M., Miura S., Nei M. (2010). Origins and evolution of microRNA genes in *Drosophila* species. Genome Biol. Evol..

[35] Li J., Liu Y., Dong D., Zhang Z. (2010). Evolution of an X-linked primate-specific micro RNA cluster. Mol. Biol. Evol..

[36] Wittbrodt J., Meyer A., Schartl M. (1998). More genes in fish?. Bioessays.

[37] Kao H., Lee S.-C. (2002). Phosphoglucose isomerases of hagfish, zebrafish, gray mullet, toad, and snake, with reference to the evolution of the genes in vertebrates. Mol. Biol. Evol..

[38] Guo B., Zou M., Wagner A. (2012). Pervasive indels and their evolutionary dynamics after the fish-specific genome duplication. Mol. Biol. Evol..

[39] Zhou X., Li Q., Lu H., Chen H., Guo Y., Cheng H., Zhou R. (2008). Fish specific duplication of Dmrt2: Characterization of zebrafish *Dmrt2b*. Biochimie.

[40] Meyer A., van de Peer Y. (2005). From 2R to 3R: Evidence for a fish-specific genome duplication (FSGD). Bioessays.

[41] Zisoulis D.G., Kai Z.S., Chang R.K., Pasquinelli A.E. (2012). Autoregulation of microRNA biogenesis by *let-7* and Argonaute. Nature.

[42] Xie G.B., Liu W.J., Pan Z.J., Cheng T.Y., Luo C. (2014). Evolution of the mir-155 family and possible targets in cancers and the immune system. Asian Pac. J. Cancer Prev..

[43] Liu S., Xia Q., Zhao P., Cheng T., Hong K., Xiang Z. (2007). Characterization and expression patterns of *let-7* microRNA in the silkworm (*Bombyx mori*). BMC Dev. Biol..

[44] Lin S., Li H., Mu H., Luo W., Li Y., Jia X., Wang S., Jia X., Nie Q., Li Y. (2012). Let-7b regulates the expression of the growth hormone receptor gene in deletion-type dwarf chickens. BMC Genom..

[45] Qin L., Chen Y., Liu X., Ye S., Yu K., Huang Z., Yu J., Zhou X., Chen H., Mo D. (2013). Integrative analysis of porcine microRNAome during skeletal muscle development. PLoS ONE.

[46] Bartel D.P. (2004). MicroRNAs: Genomics, biogenesis, mechanism, and function. Cell.

[47] Larkin M.A., Blackshields G., Brown N.P., Chenna R., McGettigan P.A., McWilliam H., Valentin F., Wallace I.M., Wilm A., Lopez R. (2007). Clustal W and Clustal X version 2.0. Bioinformatics.

[48] Zuker M. (2003). Mfold web server for nucleic acid folding and hybridization prediction. Nucleic Acids Res..

[49] Darty K., Denise A., Ponty Y. (2009). VARNA: Interactive drawing and editing of the RNA secondary structure. Bioinformatics.

[50] Tamura K., Peterson D., Peterson N., Stecher G., Nei M., Kumar S. (2011). MEGA5: Molecular evolutionary genetics analysis using maximum likelihood, evolutionary distance, and maximum parsimony methods. Mol. Biol. Evol..

[51] Lweis B.P., Shih I.H., Jones-Rhoades M.W., Bartel D.P., Burge C.B. (2003). Prediction of mammalian microRNA targets. Cell.

[52] Da Huang W., Sherman B.T., Lempicki R.A. (2009). Systematic and integrative analysis of large gene lists using DAVID bioinformatics resources. Nat. Protoc..

[53] Da Huang W., Sherman B.T., Lempicki R.A. (2009). Bioinformatics enrichment tools: Paths toward the comprehensive functional analysis of large gene lists. Nucleic Acids Res..

[54] Vandesompele J., de Preter K., Pattyn F., Poppe B., van Roy N., de Paepe A., Speleman F. (2002). Accurate normalization of real-time quantitative RT-PCR data by geometric averaging of multiple internal control genes. Genome Biol..

[55] Luo W., Zhang J., Wen J.F., Liu H., Wang W.M., Gao Z.X. (2014). Molecular cloning and expression analysis of major histocompatibility complex class I, IIA and IIB genes of blunt snout bream (*Megalobrama amblycephala*). Dev. Comp. Immunol..

