# Supplementary material for: Evolution of Fish Let-7 MicroRNAs and Their Expression Correlated to Growth Development in Blunt Snout Bream
Source: Int J Mol Sci. 2017 Mar 16;18(3):646. doi: 10.3390/ijms18030646 (PMC5372658; doi:10.3390/ijms18030646)
Supplement: Supplementary file 1 [file ijms-18-00646-s001.pdf]

Evolution of fish let-7 microRNAs and their expression correlated to growth development in blunt snout bream

Bo-Wen Zhao [1,2], Lai-Fang Zhou [1,2], Yu-Long Liu [3], Shi-Ming Wan [1,2] and Ze-Xia Gao [1,2,*]

[1] College of Fisheries, Key Lab of Agricultural Animal Genetics, Breeding and Reproduction of Ministry of Education/Key Lab of Freshwater Animal Breeding, Ministry of Agriculture, Huazhong Agricultural University, Wuhan 430070, China; zhaobowen@webmail.hzau.edu.cn (B.W.Z.); 15171617087@163.com (L.F.Z.); wansm0517@gmail.com(S.M.W.)

[2] Collaborative Innovation Center for Healthy Freshwater Aquaculture of Hubei Province, Hubei Provincial Engineering Laboratory for Pond Aquaculture, Wuhan 430070, China

[3] Department of Molecular and Cellular Biology, University of California Davis, Davis, CA 95616, USA; idrliu@ucdavis.edu

* Correspondence: gaozexia@hotmail.com; Tel./Fax: +86-27-8728-2113

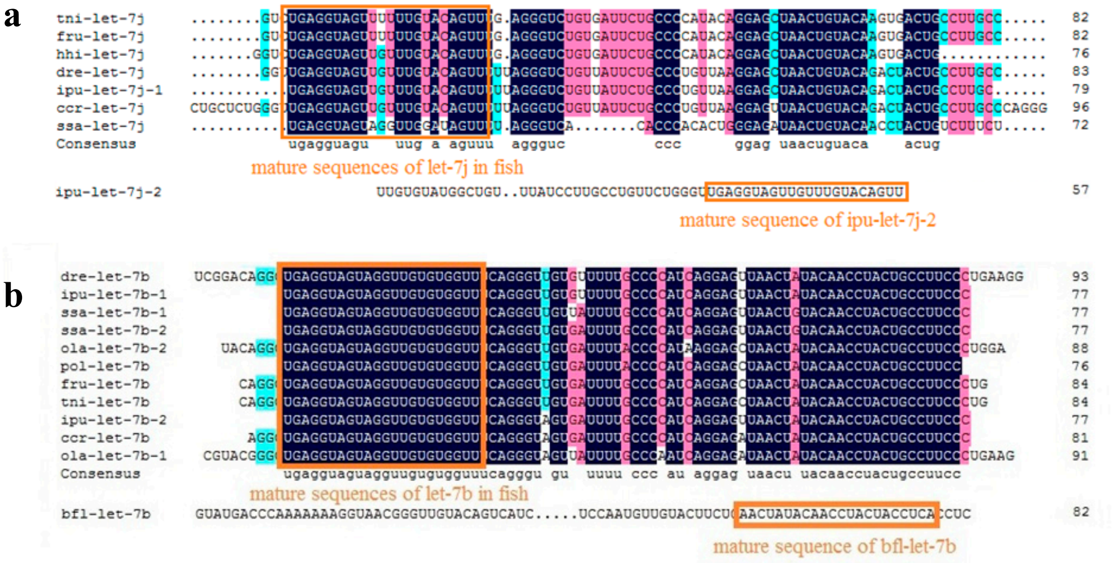

Figure S1. (a) ipu-let-7j-2 conserved sequences were located in 3'arm of precursor sequences. **(b)** bfl-let-7b conserved sequence was the reverse complementary sequence of let-7b sequence of fish species.

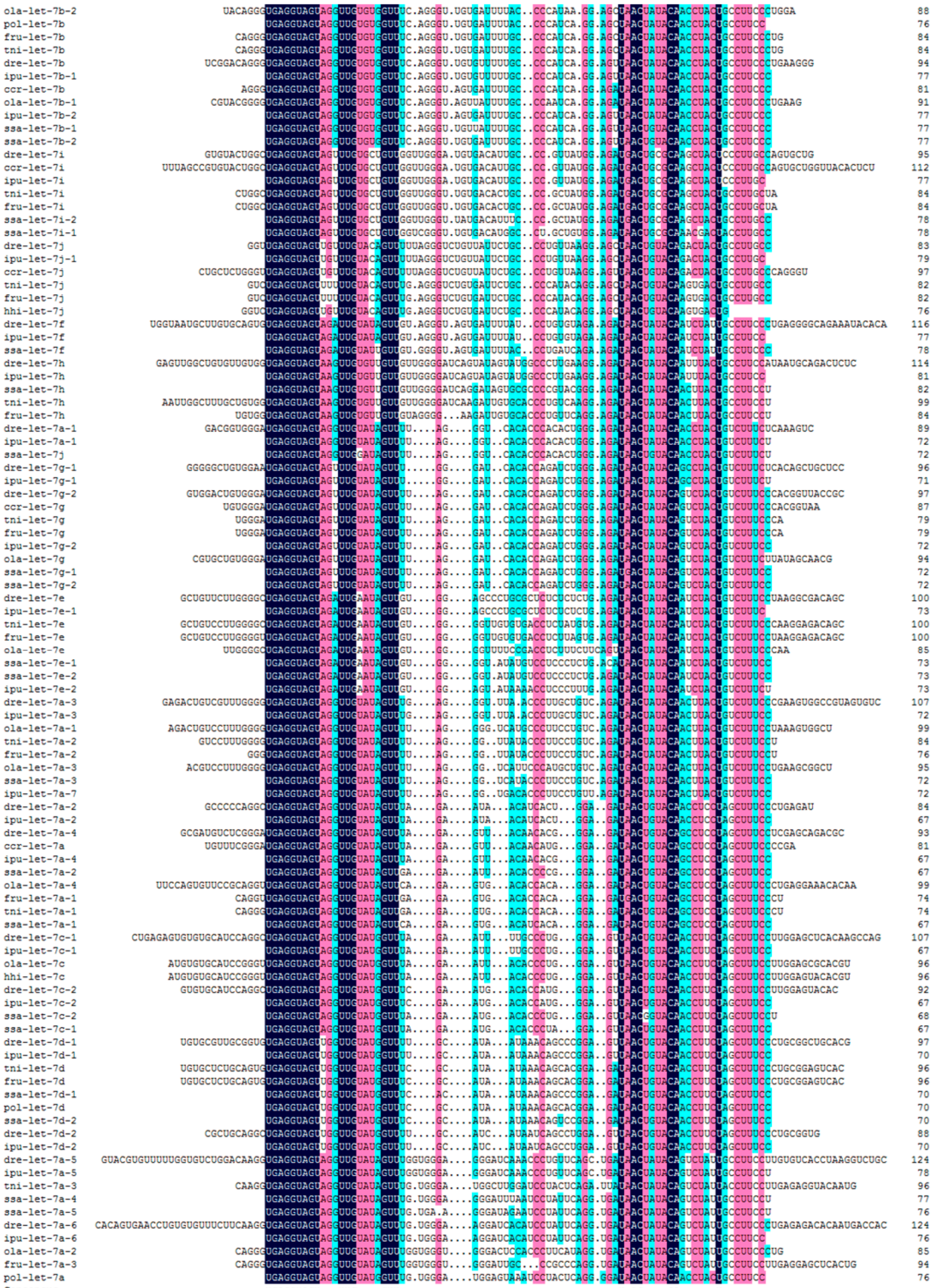

Figure S2. All fish let-7miRNAs have the same seed sequence (GAGGTAG) in 5' arm, and the let-7 miRNAs mature sequences of fish were completely conservative in three regions (TGAGGTAGT, TTG and GTT).

let-7a	dre	UGAGGUAGUAGGUUGUAUAGUU	22
	mam	UGAGGUAGUAGGUUGUAUAGUU	22
let-7b	dre	UGAGGUAGUAGGUUGUGUGGUU	22
	mam	UGAGGUAGUAGGUUGUGUGGUU	22
let-7c	dre	UGAGGUAGUAGGUUGUAUGGUU	22
	mam	UGAGGUAGUAGGUUGUAUGGUU	22
let-7d	dre	UGAGGUAGUUGGUUGUAUGGUU	22
	mam	UGAGGUAGUUGGUUGUAUGGUU	22
let-7e	dre	UGAGGUAGUAGAUUGAAUAGUU	22
	mam	UGAGGUAGUAGAUUGAAUAGUU	22
let-7f	dre	UGAGGUAGUAGAUUGUAUAGUU	22
	mam	UGAGGUAGUAGAUUGUAUAGUU	22
let-7g	dre	UGAGGUAGUAGUUUGUAUAGUU	22
	mam	UGAGGUAGUAGUUUGUAUAGUU	22
let-7h	dre	UGAGGUAGUAAGUUGUGUUGUU	22
	mam	UGAGGUAGUAAGUUGUGUUGUU	22
let-7i	dre	UGAGGUAGUAGUUUGUGCUGUU	22
	mam	UGAGGUAGUAGUUUGUGCUGUU	22
let-7j	dre	UGAGGUAGUUGUUUGUACAGUU	22
	mam	UGAGGUAGUUGUUUGUACAGUU	22

Figure S3. The sequences of let-7 miRNAs in *M. amblycephala* showed the completely consistent mature sequences with zebrafish.

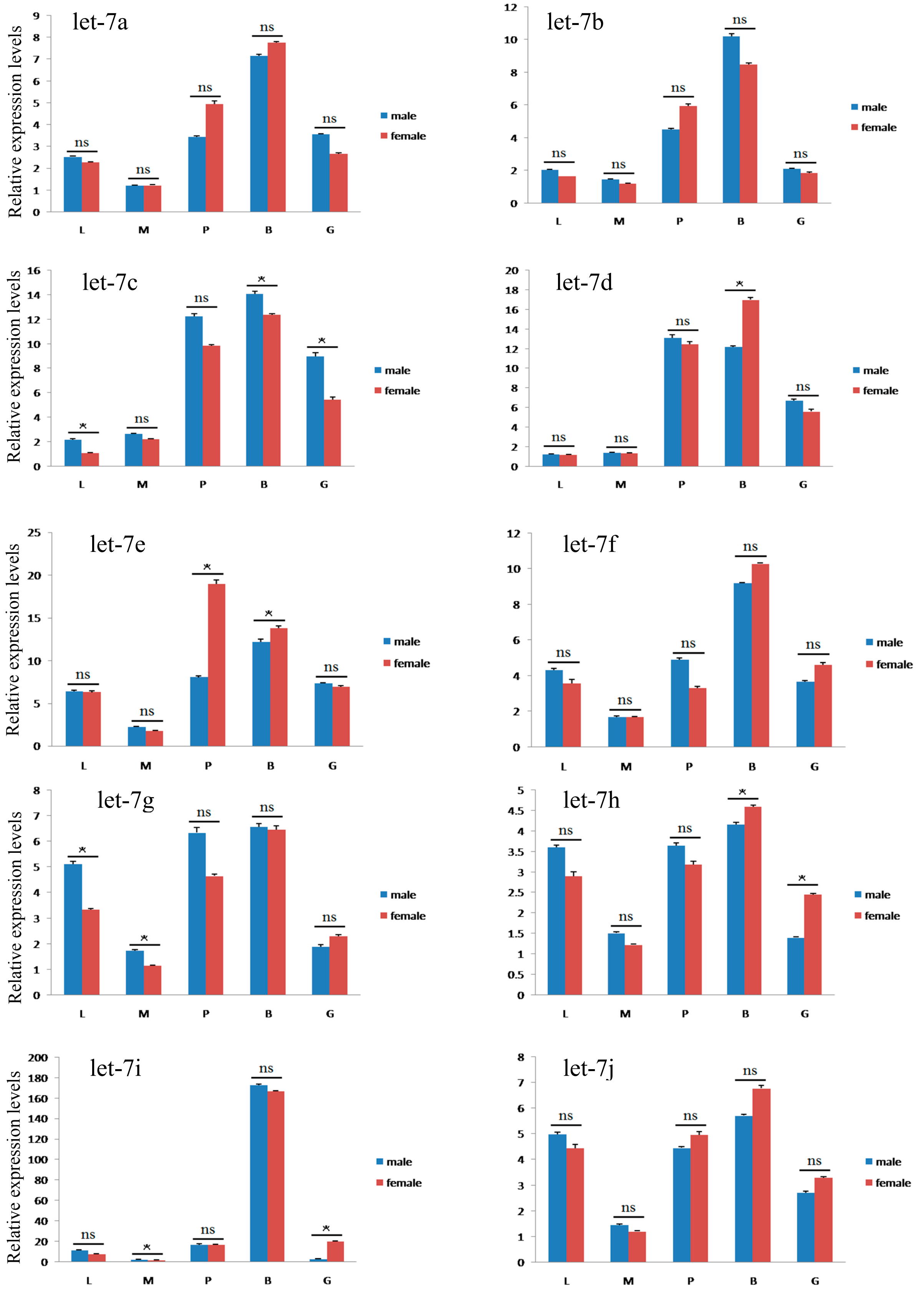

Figure S4. Let-7 miRNAs expression in five tissues of 12-month-old *M. amblycephal.* L, liver; M, muscle; P, pituitary; B, brain; G, gonad. Statistical significances between females and males are indicated as follows: $^{*}P < 0.05$; ns, not significant.

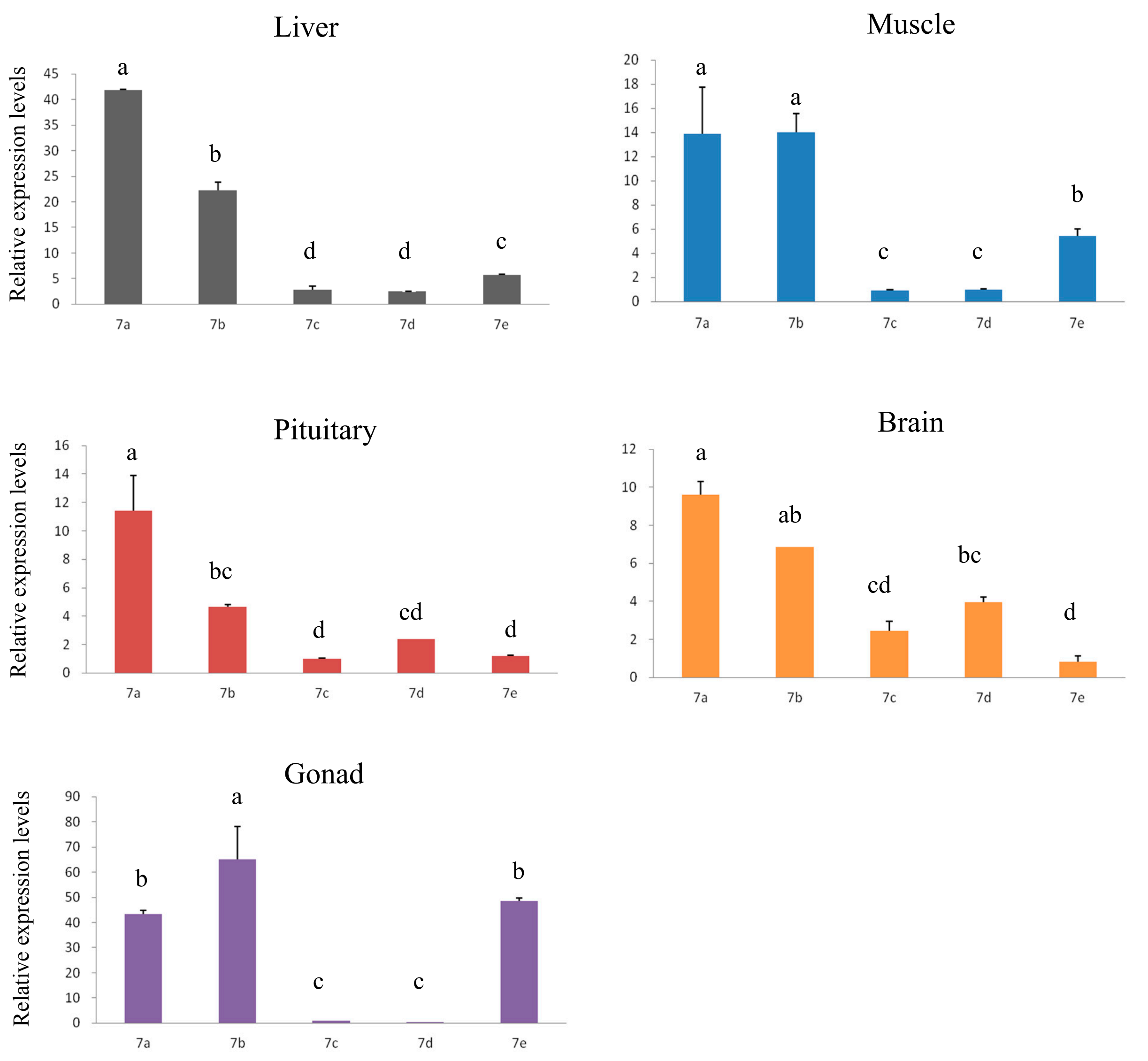

Figure S5. Let-7 miRNAs expression in five tissues of 3-month-old *M. amblycephala*. Values with the same letter mean no significant difference ($P > 0.05$).

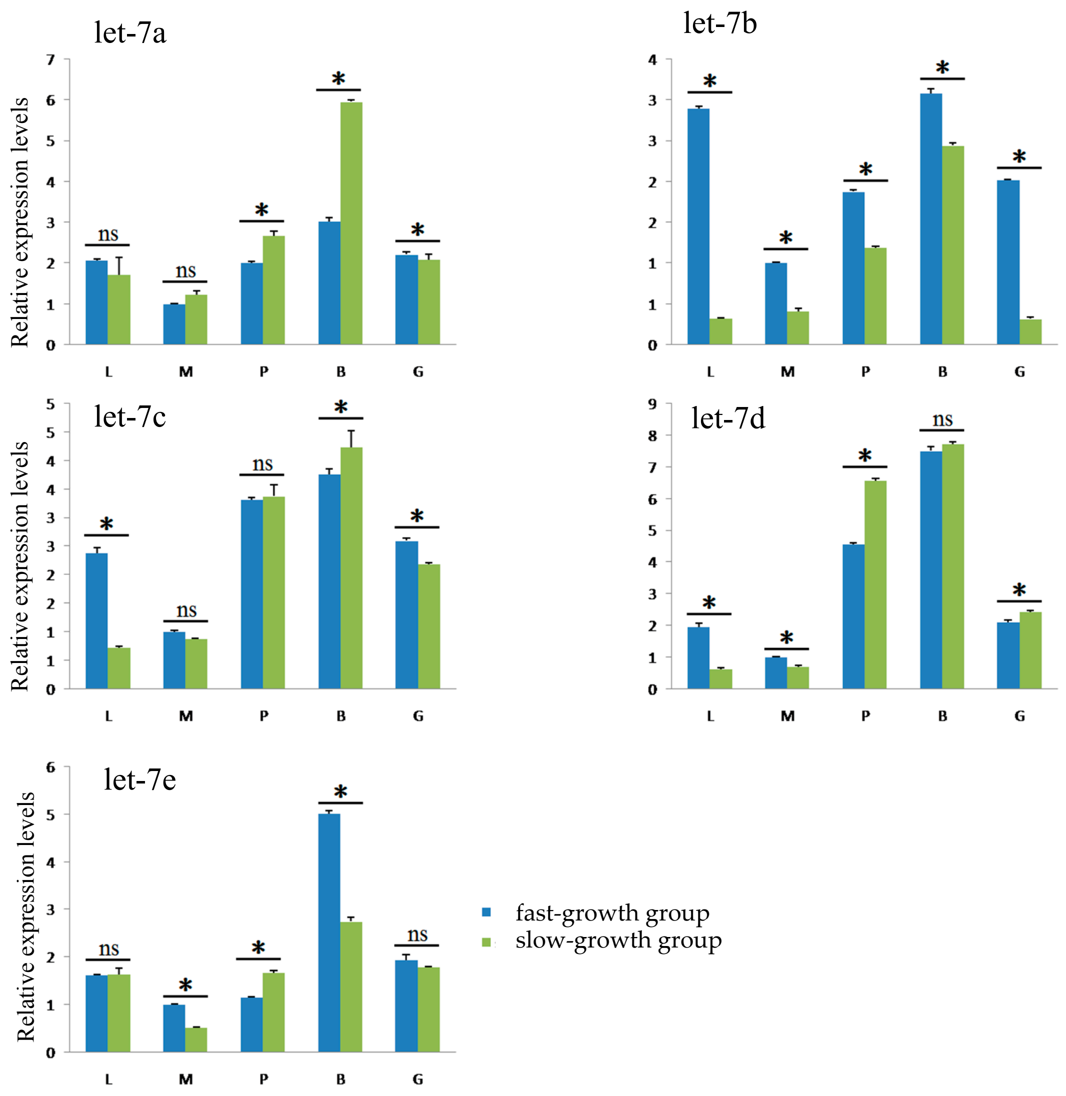

Figure S6. Let-7 miRNAs expression in five tissues of 6-month-old *M. amblycephala*. L, liver; M, muscle; P, pituitary; B, brain; G, gonad. Statistical significances between slow- and fast-growth groups are indicated as follows: *$P < 0.05$; ns, not significant.

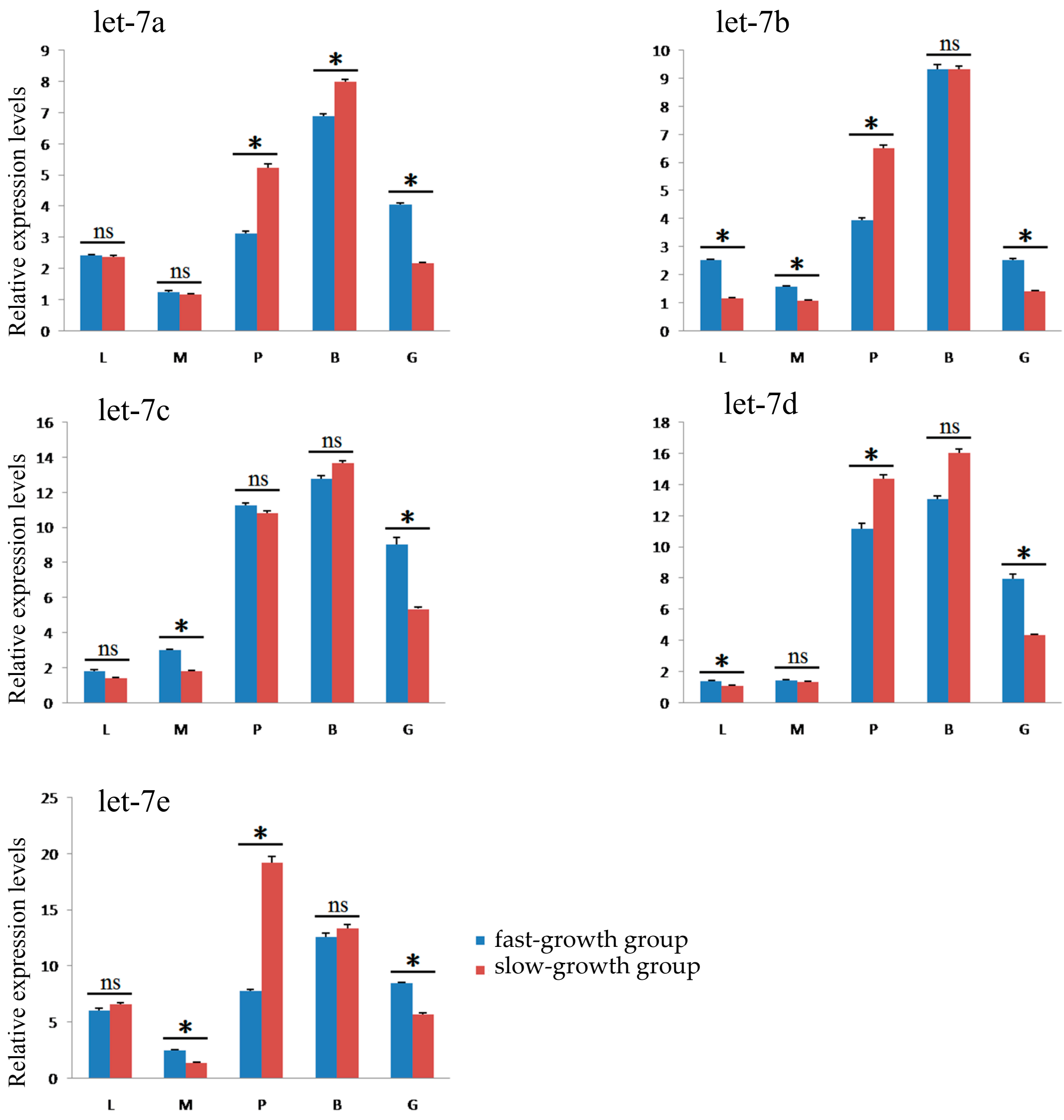

Figure S7. Let-7 miRNAs expression in five tissues of 12-month-old *M. amblycephala*. L, liver; M, muscle; P, pituitary; B, brain; G, gonad. Statistical significances between slow- and fast-growth groups are indicated as follows: *$P < 0.05$; ns, not significant.

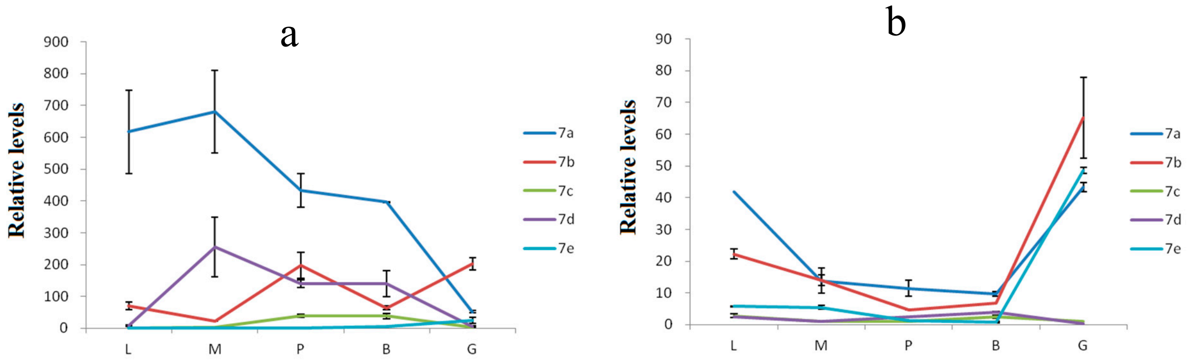

Figure S8. (a) Let-7 miRNAs expression in five tissues of 3-month-old *M. amblycephala* small size group. **(b)** Let-7 miRNAs expression in five tissues of 3-month-old *M. amblycephala* big size group. L, liver; M, muscle; P, pituitary; B, brain; G, gonad.

Table S1. All the let-7 precursor sequences of 12 fish species and 4 out-group species collected from the miRBase

Species	miRNAs	Sequences of precursor
Danio rerio	dre-let-7a-1	GACGGUGGGAUGAGGUAGUAGGUUGUAUAGUUUUAGGGUCACACCCACACUGGGAGAUAACUAUACAACCUACUGUCUUUCUCAAAG UC
	dre-let-7a-2	GCCCCCAGGCUGAGGUAGUAGGUUGUAUAGUUUAGAAUAACAUCACUGGAGAUAACUGUACAACCUCCUAGCUUUCCCUGAGAU
	dre-let-7a-3	GAGACUGUCGUUUGGGGUGAGGUAGUAGGUUGUAUAGUUUGAGGGUUUAACCCUUGCUGUCAGAUAACUAUACAACUUACUGUCUUU CCCGAAGUGGCCGUAGUGUC
	dre-let-7a-4	GCGAUGUCUCGGGAUGAGGUAGUAGGUUGUAUAGUUUAGAGUUACAACACGGGAGAUAACUGUACAGCCUCCUAGCUUUCCUCGAGC AGACGC
	dre-let-7a-5	GUACGUGUUUUUGGUGUCUGGACAAGGUGAGGUAGUAGGUUGUAUAGUUUGGUGGGAGGGAUCAAACCCUGUUCAGCUGAUAACUAU ACAGUCUAUUGCCUUCCUUGUGUCACCUAAGGUCUGC
	dre-let-7a-6	CACAGUGAACCUGUGUGUUUCUUCAAGGUGAGGUAGUAGGUUGUAUAGUUUGUGGGAAGGAUCACAUCCUAUUCAGGUGAUAACUAU ACAGUCUAUUGCCUUCCCUGAGAGACACAAUGACCACGAGUG
	dre-let-7b	UCGGACAGGGUGAGGUAGUAGGUUGUGUGGUUUCAGGGUUGUGUUUUUGCCCCAUCAGGAGUUAACUAUACAACCUACUGCCUUCCC UGAAGGG
	dre-let-7c-1	CUGAGAGUGUGUGCAUCCAGGCUGAGGUAGUAGGUUGUAUGGUUUAGAAUUUUGCCCUGGGAGUUAACUGUACAACCUUCUAGCUUU CCUUGGAGCUCACAAGCCAG
	dre-let-7c-2	GUGUGCAUCCAGGCUGAGGUAGUAGGUUGUAUGGUUUCGAAUGACACCAUGGGAGUUAACUGUACAACCUUCUAGCUUUCCUUGGAG UACAC
	dre-let-7d-1	UGUGCGUUGCGGUGUGAGGUAGUUGGUUGUAUGGUUUUGCAUAAUAAACAGCCCGGAGUUAACUGUACAACCUUCUAGCUUUCCCUG CGGCUGCACG
	dre-let-7d-2	CGCUGCAGGCUGAGGUAGUUGGUUGUAUGGUUUUGCAUCAUAAUCAGCCUGGAGUUAACUGUACAACCUUCUAGCUUUCCCUGCGGUG
	dre-let-7e	GCUGUUCUUGGGGCUGAGGUAGUAGAUUGAAUAGUUGUGGAGCCCUGCGCUCUCUCUCUGAGAUAACUAUACAAUCUACUGUCUUUCC UAAGGCGACAGC
	dre-let-7f	UGGUAAUGCUUGUGCAGUGUGAGGUAGUAGAUUGUAUAGUUGUAGGGUAGUGAUUUUAUCCUGUGUAGAAGAUAACUAUACAAUCUA UUGCCUUCCCUGAGGGGCAGAAAUACACA
	dre-let-7g-1	GGGGGCUGUGGAAUGAGGUAGUAGUUUGUAUAGUUUGGGAUCACACCAGAUCUGGGAGAUAACUAUACAGCCUACUGUCUUUCUCAC AGCUGCUCC
	dre-let-7g-2	GUGGACUGUGGGAUGAGGUAGUAGUUUGUAUAGUUUUAGGAUCACACCAGAUCUGGGAGAUAACUAUACAGUCUACUGUCUUUCCCA CGGUUACCGC
	dre-let-7h	GAGUUGGCUGUGUUGUGGUGAGGUAGUAAGUUGUGUUGUUGUUGGGGAUCAGUAUAGUAUGGCCCUUGAAGGAGAUAACUAUACAAU UUACUGCCUUCCAUAAUGCAGACUCUC
	dre-let-7i	GUGUACUGGCUGAGGUAGUAGUUUGUGCUGUUGGUUGGGAUGUGACAUUGCCCGUUAUGGAGAUGACUGCGCAAGCUACUCCCUUGC CAGUGCUG
	dre-let-7j	GGUUGAGGUAGUUGUUUGUACAGUUUUUAGGGUCUGUUAUUCUGCCCUGUUAAGGAGCUAACUGUACAGACUACUGCCUUGCC

Oryzias latipes	ola-let-7a-1	AGACUGUCCUUUGGGGUGAGGUAGUAGGUUGUAUAGUUUUAGGGGUCAUGCCCUUCCUGUCAGAUAACUAUACAACUUACUGUCUUU CCUAAAGUGGCU
	ola-let-7a-2	CAGGGUGAGGUAGUAGGUUGUAUAGUUUGGUGGGUGGGACUCCACCCUUCAUAGGUGAUAACUAUACAGUCUAUUGCCUUCCCUG
	ola-let-7a-3	ACGUCCUUUGGGGUGAGGUAGUAGGUUGUAUAGUUUUAGGGUCAUUCCCAUGCUGUCAGAUGACUAUACAACUUACUGUCUUUCCUG AAGCGGCU
	ola-let-7a-4	UUCCAGUGUUCCGCAGGUUGAGGUAGUAGGUUGUAUAGUUCAGAGUGACACCACAGGAGAUAACUGUACAGCCUCCUAGCUUUCCCUG AGGAAACACAA
	ola-let-7b-1	CGUACGGGGUGAGGUAGUAGGUUGUGUGGUUUCAGGGUAGUUAUUUUGCCCAAUCAGGAGAUAACUAUACAACCUACUGCCUUCCCU GAAG
	ola-let-7b-2	UACAGGGUGAGGUAGUAGGUUGUGUGGUUUCAGGGUUGUGAUUUUACCCCAUAAGGAGCUAACUAUACAACCUACUGCCUUCCCUGG A
	ola-let-7c	AUGUGUGCAUCCGGGUUGAGGUAGUAGGUUGUAUGGUUUAGAAUUACACCCUGGGAGUUAACUGUACAACCUUCUAGCUUUCCUUGG AGCGCACGU
	ola-let-7e	UUGGGGCUGAGGUAGUAGAUUGAAUAGUUGUGGGGUUUUCCGACCUCUUUCUUCAGUUAACUAUACAAUCUACUGUCUUUCCCAA
	ola-let-7g	CGUGCUGUGGGAUGAGGUAGUAGUUUGUAUAGUUUUAGGAUCACACCAGAUCUGGGAGAUAACUAUACAGUCUACUGUCUUUCUUAU AGCAACG
Fugu rubripes	fru-let-7a-1	CAGGUUGAGGUAGUAGGUUGUAUAGUUGAGAGUGACACCACAGGAGAUGACUGUACAGCCUCCUAGCUUUCCCU
	fru-let-7a-2	GGGUGAGGUAGUAGGUUGUAUAGUUUUAGGGUUAUACCCUUCCUGUCAGAUAACUAUACAACUUACUGUCUUUCCU
	fru-let-7a -3	CAGGGUGAGGUAGUAGGUUGUAUAGUUUGGUGGGUGGGAUUGCCCGCCCAGGUGAUAACUAUACAGUCUAUUGCCUUCCUUGAGGAG CUCACUG
	fru-let-7b	CAGGGUGAGGUAGUAGGUUGUGUGGUUUCAGGGUUGUGAUUUUGCCCCAUCAGGAGCUAACUAUACAACCUACUGCCUUCCCUG
	fru-let-7d	UGUGCUCUGCAGUGUGAGGUAGUUGGUUGUAUGGUUUCGCAUAAUAAACAGCACGGAGAUAACUGUACAACCUUCUAGCUUUCCCUG CGGAGUCAC
	fru-let-7e	GCUGUCCUUGGGGUUGAGGUAGUAGAUUGAAUAGUUGUGGGGUUGUGUGACCUCUUAGUGAGAUAACUAUACAAUCUACUGUCUUUC CUAAGGAGACAGC
	fru-let-7g	UGGGAUGAGGUAGUAGUUUGUAUAGUUUUAGGAUCACACCAGAUCUGGGAGAUAACUAUACAGUCUACUGUCUUUCCCA
	fru-let-7h	UGUGGUGAGGUAGUAAGUUGUGUUGUUGUAGGGGAAGAUUGUGCACCCUGUUCAGGAGAUAACUAUACAACUUACUGCCUUCCU
	fru-let-7i	CUGGCUGAGGUAGUAGUUUGUGCUGUUGGUUGGGUUGUGACACUGCCCGCUAUGGAGAUGACUGCGCAAGCUACUGCCUUGCUA
	fru-let-7j	GUCUGAGGUAGUUUUUUGUACAGUUUGAGGGUCUGUGAUUCUGCCCCAUACAGGAGCUAACUGUACAAGUGACUGCCUUGCC
	tni-let-7a-1	CAGGGUGAGGUAGUAGGUUGUAUAGUUGAGAGUGACACCACAGGAGAUAACUGUACAGCCUCCUAGCUUUCCCU

Tetraodon nigroviridis	tni-let-7a-2	GUCCUUUGGGGUGAGGUAGUAGGUUGUAUAGUUUUAGGGUUAUACCCUUCCUGUCAGAUAACUAUACAACUUACUGUCUUUCCU
	tni-let-7a-3	CAAGGUGAGGUAGUAGGUUGUAUAGUUUGUGGGAUGGCUUGGAUCCUACUCAGAUUAUAACUAUACAGUCUAUUACCUUCCUUGAGA GGUACAAUG
	tni-let-7b	CAGGGUGAGGUAGUAGGUUGUGUGGUUUCAGGGUUGUGAUUUUGCCCCAUCAGGAGCUAACUAUACAACCUACUGCCUUCCCUG
	tni-let-7d	UGUGCUCUGCAGUGUGAGGUAGUUGGUUGUAUGGUUUCGCAUAAUAAACAGCACGGAGAUAACUGUACAACCUUCUAGCUUUCCCUG CGGAGUCAC
	tni-let-7e	GCUGUCCUUGGGGCUGAGGUAGUAGAUUGAAUAGUUGUGGGGUUGUGUGACCUCUAUGUGAGAUAACUAUACAAUCUACUGUCUUUC CCAAGGAGACAGC
	tni-let-7g	UGGGAUGAGGUAGUAGUUUGUAUAGUUUUAGGAUCACACCAGAUCUGGGAGAUAACUAUACAGUCUACUGUCUUUCCCA
	tni-let-7h	AAUUGGCUUUGCUGUGGUGAGGUAGUAAGUUGUGUUGUUGUUGGGGAUCAAGAUUGUGCACCCUGUCAAGGAGAUAACUAUACAACU UACUGCCUUCCU
	tni-let-7i	CUGGCUGAGGUAGUAGUUUGUGCUGUUGGUUGGGUUGUGACACUGCCCGCUAUGGAGAUGACUGCGCAAGCUACUGCCUUGCUA
	tni-let-7j	GUCUGAGGUAGUUUUUUGUACAGUUUGAGGGUCUGUGAUUCUGCCCCAUACAGGAGCUAACUGUACAAGUGACUGCCUUGCC
Ictalurus punctatus	ipu-let-7a-1	UGAGGUAGUAGGUUGUAUAGUUUUAGGGUCACACCCACACUGGGAGAUAACUAUACAACCUACUGUCUUUCU
	ipu-let-7a-2	UGAGGUAGUAGGUUGUAUAGUUUAGAAUAACAUCACUGGAGAUAACUGUACAACCUCCUAGCUUUCC
	ipu-let-7a-3	UGAGGUAGUAGGUUGUAUAGUUUGAGGGUUUAACCCUUGCUGUCAGAUAACUAUACAACUUACUGUCUUUCC
	ipu-let-7a-4	UGAGGUAGUAGGUUGUAUAGUUUAGAGUUACAACACGGGAGAUAACUGUACAGCCUCCUAGCUUUCC
	ipu-let-7a-5	UGAGGUAGUAGGUUGUAUAGUUUGGUGGGAGGGAUCAAACCCUGUUCAGCUGAUAACUAUACAGUCUAUUGCCUUCCU
	ipu-let-7a-6	UGAGGUAGUAGGUUGUAUAGUUUGUGGGAAGGAUCACAUCCUAUUCAGGUGAUAACUAUACAGUCUAUUGCCUUCC
	ipu-let-7a-7	UGAGGUAGUAGGUUGUAUAGUUUUAGGGUGACACCCUUCCUGUUAGAUAACUAUACAACUUACUGUCUUUCC
	ipu-let-7b-1	UGAGGUAGUAGGUUGUGUGGUUUCAGGGUUGUGUUUUUGCCCCAUCAGGAGUUAACUAUACAACCUACUGCCUUCCC
	ipu-let-7b-2	UGAGGUAGUAGGUUGUGUGGUUUCAGGGUAGUGAUUUUGCCCCAUCAGGAGUUAACUAUACAACCUACUGCCUUCCC
	ipu-let-7c-1	UGAGGUAGUAGGUUGUAUGGUUUAGAAUUUUGCCCUGGGAGUUAACUGUACAACCUUCUAGCUUUCC
	ipu-let-7c-2	UGAGGUAGUAGGUUGUAUGGUUUCGAAUGACACCAUGGGAGUUAACUGUACAACCUUCUAGCUUUCC
	ipu-let-7d-1	UGAGGUAGUUGGUUGUAUGGUUUUGCAUAAUAAACAGCCCGGAGUUAACUGUACAACCUUCUAGCUUUCC

	ipu-let-7d-2	UGAGGUAGUUGGUUGUAUGGUUUUGCAUCAUAAUCAGCCUGGAGUUAACUGUACAACCUUCUAGCUUUCC
	ipu-let-7e-1	UGAGGUAGUAGAUUGAAUAGUUGUGGAGCCCUGCGCUCUCUCUCUGAGAUAACUAUACAAUCUACUGUCUUUC
	ipu-let-7e-2	UGAGGUAGUAGAUUGAAUAGUUGUGGAGUAUAAAACCUCCCUUUGAGAUAACUAUACAAUCUACUGUCUUUCU
	ipu-let-7f	UGAGGUAGUAGAUUGUAUAGUUGUAGGGUAGUGAUUUUAUCCUGUGUAGAAGAUAACUAUACAAUCUAUUGCCUUCC
	ipu-let-7g-1	UGAGGUAGUAGUUUGUAUAGUUUGGGAUCACACCAGAUCUGGGAGAUAACUAUACAGCCUACUGUCUUUCU
	ipu-let-7g-2	UGAGGUAGUAGUUUGUAUAGUUUUAGGAUCACACCAGAUCUGGGAGAUAACUAUACAGUCUACUGUCUUUCC
	ipu-let-7h	UGAGGUAGUAAGUUGUGUUGUUGUUGGGGAUCAGUAUAGUAUGGCCCUUGAAGGAGAUAACUAUACAAUUUACUGCCUUCC
	ipu-let-7i	UGAGGUAGUAGUUUGUGCUGUUGGUUGGGAUGUGACAUUGCCCGUUAUGGAGAUGACUGCGCAAGCUACUCCCUUG
	ipu-let-7j-1	UGAGGUAGUUGUUUGUACAGUUUUUAGGGUCUGUUAUUCUGCCCUGUUAAGGAGCUAACUGUACAGACUACUGCCUUGC
	ipu-let-7j-2	UUGUGUAUGGCUGUUUAUCCUUGCCUGUUCUGGGUUGAGGUAGUUGUUUGUACAGUU
Cyprinus carpio	ccr-let-7a	UGUUUCGGGAUGAGGUAGUAGGUUGUAUAGUUUAGAGUUACAACAUGGGAGAUAACUGUACAGCCUCCUAGCUUUCCCCGA
	ccr-let-7b	AGGGUGAGGUAGUAGGUUGUGUGGUUUCAGGGUAGUGAUUUUGCCCCAUCAGGAGAUAACUAUACAACCUACUGCCUUCCC
	ccr-let-7g	UGUGGGAUGAGGUAGUAGUUUGUAUAGUUUUAGGAUCACACCAGAUCUGGGAGAUAACUAUACAGUCUACUGUCUUUCCCACGGUAA
	ccr-let-7i	UUUAGCCGUGUACUGGCUGAGGUAGUAGUUUGUGCUGUUGGUUGGGAUGUGACAUUGCCCGUUAUGGAGAUGACUGCGCAAGCUACU<br>CCCUUGCCAGUGCUGGUUACACUCU
	ccr-let-7j	CUGCUCUGGGUUGAGGUAGUUGUUUGUACAGUUUUUAGGGUCUGUUAUUCUGCCCUGUUAAGGAGUUAACUGUACAGACUACUGCCU<br>UGCCCAGGGU
Paralichthys olivaceus	pol-let-7a	UGAGGUAGUAGGUUGUAUGGUUUGUGGGAUGGAGUAAAUCCUACUCAGGGGAUAACUAUACAACCUACUGCCUUCC
	pol-let-7b	UGAGGUAGUAGGUUGUGUGGUUUCAGGGUUGUGAUUUUACCCCAUCAGGAGCUAACUAUACAACCUACUGCCUUCC
	pol-let-7d	UGAGGUAGUUGGUUGUAUGGUUUCGCAUAAUAAACAGCACGGAGAUAACUGUACAACCUUCUAGCUUUCC
Hippoglossus hippoglossus	hhi-let-7c	AUGUGUGCAUCCGGGUUGAGGUAGUAGGUUGUAUGGUUUAGAAUUACACCCUGGGAGUUAACUGUACAACCUUCUAGCUUUCCUUGG<br>AGUACACGU
	hhi-let-7j	GGUCUGAGGUAGUUGUUUGUACAGUUUGAGGGUCUGUGAUUCUGCCCCAUACAGGAGCUAACUGUACAAGUGACUG
Salmo salar	ssa-let-7a-1	UGAGGUAGUAGGUUGUAUAGUUCAGAGUGACAUCACAGGAGAUAACUGUACAGCCUCCUAGCUUUCC

	ssa-let-7a-2	UGAGGUAGUAGGUUGUAUAGUUGAGAAUUACACCCCGGGAGAUAACUGUACAGCCUCCUAGCUUUCC
	ssa-let-7a-3	UGAGGUAGUAGGUUGUAUAGUUUUAGGGUCAUACCCUUCCUGUCAGAUAACUAUACAACUUACUGUCUUUCC
	ssa-let-7a-4	UGAGGUAGUAGGUUGUAUAGUUUGUGGGAGGGAUUUAAUCCUAUUCAGGUGAUAACUAUACAGUCUAUUGCCUUCCU
	ssa-let-7a-5	UGAGGUAGUAGGUUGUAUAGUUUGUGAAGGGAUAGAAUCCUAUUCAGGUGAUAACUAUACAGUCUAUUGCCUUCCU
	ssa-let-7b-1	UGAGGUAGUAGGUUGUGUGGUUUCAGGGUUGUUAUUUUGCCCCAUCAGGAGUUAACUGUACAACCUACUGCCUUCCC
	ssa-let-7b-2	UGAGGUAGUAGGUUGUGUGGUUUCAGGGUUGUGAUUUUGCCCCAUCAGGAGUUAACUGUACAACCUACUGCCUUCCC
	ssa-let-7c-1	UGAGGUAGUAGGUUGUAUGGUUUAGAAUGACACCCUAGGAGUUAACUGUACAACCUUCUAGCUUUCC
	ssa-let-7c-2	UGAGGUAGUAGGUUGUAUGGUUUAGAAUGACACCCUGGGAGUUAACGGUACAACCUUCUAGCUUUCCU
	ssa-let-7d-1	UGAGGUAGUUGGUUGUAUGGUUUCACAUAAUAAACAGCCCGGAGAUAACUGUACAACCUUCUAGCUUUCC
	ssa-let-7d-2	UGAGGUAGUUGGUUGUAUGGUUUCGCAUAAUAAACAGUCCGGAGAUAACUGUACAACCUUCUAGCUUUCC
	ssa-let-7e-1	UGAGGUAGUAGAUUGAAUAGUUGUGGGGUAUAUGUCCUCCCUCUGACAUAACUAUACAAUCUACUGUCUUUCC
	ssa-let-7e-2	UGAGGUAGUAGAUUGAAUAGUUGUGGGGUAUAUGUCCUCCCUCUGAGAUAACUAUACAAUCUACUGUCUUUCC
	ssa-let-7f	UGAGGUAGUAGAUUGUAUUGUUGUGGGGUAGUGAUUUUACCCUGAUCAGAAGAUAACUAUACAAUCUAUUGCCUUCCC
	ssa-let-7g-1	UGAGGUAGUAGUUUGUAUAGUUUUAGGAUCACACCAGAUCUGGGAGAUGACUAUACAGUCUACUGUCUUUCC
	ssa-let-7g-2	UGAGGUAGUAGUUUGUAUAGUUUUAGGAUCACACCAGAUCUGGGAGAUAACUAUACAGUCUACUGUCUUUCC
	ssa-let-7h	UGAGGUAGUAAGUUGUGUUGUUGUUGGGGAUCAGGAUAGUGCGCCCCGUACGGGAGAUAACUAUACAACUUACUGCCUUCCU
	ssa-let-7i-1	UGAGGUAGUAGUUUGUGCUGUUGGUCGGGUUGUGACAUGGCCUGCUGUGGAGAUAACUGCGCAAACGACUACCUUGCC
	ssa-let-7i-2	UGAGGUAGUAGUUUGUGCUGUUGGUUGGGUUAUGACAUUUCCCGCUAUGGAGAUGACUGCGCAAGCUACUGCCUUGCC
	ssa-let-7j	UGAGGUAGUAGGUUGGAUAGUUUUAGGGUCACACCCACACUGGGAGAUAACUGUACAACCUACUGUCUUUCU
Branchiostoma floridae	bfl-let-7a-1	CUGAGGUGAGGUAGUAGGUUGUAUAGUUCAGAAGUACAACAUUGGAGAUGACUGUACAACCCGUUACCUUUUUUUGGGUCAU
	bfl-let-7a-2	AGACACGGGUGCACCUGAGGUGAGGUAGUAGGUUGUAUAGUUGAGAAGUACAUCAUUGGAGAUAACUGUGCAACCUGCUAGCUCUCC UUGGGGCAUGCC
	bfl-let-7b	GUAUGACCCAAAAAAAGGUAACGGGUUGUACAGUCAUCUCCAAUGUUGUACUUCUGAACUAUACAACCUACUACCUCACCUC

Branchiostoma belcheri	bbe-let-7a-1	CUGAGGUGAGGUAGUAGGUUGUAUAGUUGAGAAGUACAACAUUGGAGAUGACUGUACAACCCGUUACCUUUUUUCGGGUCAU
	bbe-let-7a-2	AGACAAGGGUGCGCCUGAGGUGAGGUAGUAGGUUGUAUAGUUGAGAAGUACAUCAUUGGAGAUAACUGUGCAACCUGCUAGCUCUCC UUGGGGCAUGCC
Homo sapiens	hsa-let-7a-1	UGGGAUGAGGUAGUAGGUUGUAUAGUUUUAGGGUCACACCCACCACUGGGAGAUAACUAUACAAUCUACUGUCUUUCCUA
	hsa-let-7a-2	AGGUUGAGGUAGUAGGUUGUAUAGUUUAGAAUUACAUCAAGGGAGAUAACUGUACAGCCUCCUAGCUUUCCU
	hsa-let-7a-3	GGGUGAGGUAGUAGGUUGUAUAGUUUGGGGCUCUGCCCUGCUAUGGGAUAACUAUACAAUCUACUGUCUUUCCU
	hsa-let-7b	CGGGGUGAGGUAGUAGGUUGUGUGGUUUCAGGGCAGUGAUGUUGCCCCUCGGAAGAUAACUAUACAACCUACUGCCUUCCCUG
	hsa-let-7c	GCAUCCGGGUUGAGGUAGUAGGUUGUAUGGUUUAGAGUUACACCCUGGGAGUUAACUGUACAACCUUCUAGCUUUCCUUGGAGC
	hsa-let-7d	CCUAGGAAGAGGUAGUAGGUUGCAUAGUUUUAGGGCAGGGAUUUUGCCCACAAGGAGGUAACUAUACGACCUGCUGCCUUUCUUAGG
	hsa-let-7e	CCCGGGCUGAGGUAGGAGGUUGUAUAGUUGAGGAGGACACCCAAGGAGAUCACUAUACGGCCUCCUAGCUUUCCCCAGG
	hsa-let-7f-1	UCAGAGUGAGGUAGUAGAUUGUAUAGUUGUGGGGUAGUGAUUUUACCCUGUUCAGGAGAUAACUAUACAAUCUAUUGCCUUCCCUGA
	hsa-let-7f-2	UGUGGGAUGAGGUAGUAGAUUGUAUAGUUUUAGGGUCAUACCCCAUCUUGGAGAUAACUAUACAGUCUACUGUCUUUCCCACG
	hsa-let-7g	AGGCUGAGGUAGUAGUUUGUACAGUUUGAGGGUCUAUGAUACCACCCGGUACAGGAGAUAACUGUACAGGCCACUGCCUUGCCA
	hsa-let-7i	CUGGCUGAGGUAGUAGUUUGUGCUGUUGGUCGGGUUGUGACAUUGCCCGCUGUGGAGAUAACUGCGCAAGCUACUGCCUUGCUA
Mus musculus	mmu-let-7a-1	UUCACUGUGGGAUGAGGUAGUAGGUUGUAUAGUUUUAGGGUCACACCCACCACUGGGAGAUAACUAUACAAUCUACUGUCUUUCCUA AGGUGAU
	mmu-let-7a-2	CUGCAUGUUCCCAGGUUGAGGUAGUAGGUUGUAUAGUUUAGAGUUACAUCAAGGGAGAUAACUGUACAGCCUCCUAGCUUUCCUUGG GACUUGCAC
	mmu-let-7b	GCAGGGUGAGGUAGUAGGUUGUGUGGUUUCAGGGCAGUGAUGUUGCCCCUCCGAAGAUAACUAUACAACCUACUGCCUUCCCUGA
	mmu-let-7c-1	UGUGUGCAUCCGGGUUGAGGUAGUAGGUUGUAUGGUUUAGAGUUACACCCUGGGAGUUAACUGUACAACCUUCUAGCUUUCCUUGGA GCACACU
	mmu-let-7c-2	ACGGCCUUUGGGGUGAGGUAGUAGGUUGUAUGGUUUUGGGCUCUGCCCCGCUCUGCGGUAACUAUACAAUCUACUGUCUUUCCUGAAG UGGCCGC
	mmu-let-7e	CGCGCCCCCCGGGCUGAGGUAGGAGGUUGUAUAGUUGAGGAAGACACCCGAGGAGAUCACUAUACGGCCUCCUAGCUUUCCCCAGGCU GCGCC
	mmu-let-7f-1	AUCAGAGUGAGGUAGUAGAUUGUAUAGUUGUGGGGUAGUGAUUUUACCCUGUUUAGGAGAUAACUAUACAAUCUAUUGCCUUCCCUG AG
	mmu-let-7f-2	UGUGGGAUGAGGUAGUAGAUUGUAUAGUUUUAGGGUCAUACCCCAUCUUGGAGAUAACUAUACAGUCUACUGUCUUUCCCACG

	mmu-let-7g	CCAGGCUGAGGUAGUAGUUUGUACAGUUUGAGGGUCUAUGAUACCACCCGGUACAGGAGAUAACUGUACAGGCCACUGCCUUGCCAGG
	mmu-let-7i	CUGGCUGAGGUAGUAGUUUGUGCUGUUGGUCGGGUUGUGACAUUGCCCGCUGUGGAGAUAACUGCGCAAGCUACUGCCUUGCUAG
	mmu-let-7j	AUUGGAGGCUAUUUCUGAUCAUGAUAAUUUCCUGAGGUAUUAGUUUGUGCUGUUAUAUGAAUCGAAUAAUAUCCCUUGCUCAGAUUA AAAGCCUGGAGUUAAAAAAAUCAAGUGCCUUGAAC
	mmu-let-7k	UAGCCACAGCCCUAACCCUAGCCUGAGGUAGGAGGUUGUGUGCAAGCUCACCACUAACCUAUAGUACACAGAGAGCCUUUAUCCCACA ACACAACACAAAACAAUACUUC
Petromyzon marinus	pma-let-7a-1	UGUGGGCUCCUGGGUUGAGGUAGUAGGUUGUAUAGUUUAAGGGGCAGACAUUCAUCUUCCCUUAUCGGAGAUAAGCUAUGCAGCCAA CUGCCUUUCCCAGGGCUUCGC
	pma-let-7a-2	UGAGUGCGCCCUGGGAUGAGGUAGUAGGUUGUAUAGUUUCAGGGUCACACCCAAACUGGGAGUUAACUGUACAAUCUACUCUCUUUCC CAAGGCGGCACGCUG
	pma-let-7a-3	GCGUGGGAUGAGGUAGUAGGUUGUAUAGUUUGGGGAGUGUGACCCCGUUCAGGAGAUAACUGUACAAUCUACUGUCUUGCCCAAGC
	pma-let-7a-4	GUUUUCGGGGUGAGGUAGUAGGUUGUAUAGUUUAGCAUUACACCUACGGAGAUAACUGUACAACCUUCUAGCUUUCCCCGCGGGC
	pma-let-7b	GCAGGGUGAGGUAGUAGGUUUUGUAGUUCUGCGGGCAGAGAUUUUGCCCAGCGAGUGGAGAUGAACUAUAUAAUCUACUGCCUUGCC CGGA
	pma-let-7c	CCGGCUCGCGGGGUGAGGUAGUAGAUUGUAUGGUUUUAGGGUCACACCCUCGGUUGGGAGAUAACUAUGCAAUCUACUGUCUUUCCCG CGGGUGCG
	pma-let-7d	GGUGAGGUGAGGUAGUAGGUUGUAAAGUUGUAGGGUGUCAUUUGUUUACACCCGAUUUGGAGAUAACUGUACAACCUAUUGCCUUC
Drosophila melanogaster	dme-let-7	UCUGGCAAAUUGAGGUAGUAGGUUGUAUAGUAGUAAUUACACAUCAUACUAUACAAUGUGCUAGCUUUCUUUGCUUGA
Caenorhabditis elegans	cel-let-7	UACACUGUGGAUCCGGUGAGGUAGUAGGUUGUAUAGUUUGGAAUAUUACCACCGGUGAACUAUGCAAUUUUCUACCUUACCGGAGAC AGAACUCUUCGA

Table S2 The phenotypic data of sampling fish in fast- and slow-growth groups of *Megalobrama amblycephala*

Groups	3-month old			6-month old			12-month old		
	Body length (cm)	Body height (cm)	Body weight (g)	Body length (cm)	Body height (cm)	Body weight (g)	Body length (cm)	Body height (cm)	Body weight (g)
Fast-growth group	9.3	3.6	15.2	12.2	4.9	41.7	13.0	5.2	48.6
	9.6	3.6	16.5	12.1	4.8	38.2	13.3	5.5	51.1
	9.2	3.6	16.0	11.7	4.6	36.4	13.1	5.0	44.7
Slow-growth group	5.0	1.8	3.1	5.9	2.0	3.6	7.5	2.5	7.2
	5.3	1.8	3.6	6.5	2.2	4.8	7.5	2.6	7.7
	5.1	1.8	2.9	6.4	2.3	5.4	8.2	3.0	10.6
